# Electrocatalytic activity and surface oxide reconstruction of bimetallic iron–cobalt nanocarbide electrocatalysts for the oxygen evolution reaction†

**DOI:** 10.1039/d3ra07003d

**Published:** 2023-11-14

**Authors:** Amanda J. Ritz, Isabella A. Bertini, Edward T. Nguyen, Geoffrey F. Strouse, Robert A. Lazenby

**Affiliations:** a Department of Chemistry & Biochemistry, Florida State University Tallahassee Florida 32306 USA rlazenby@fsu.edu

## Abstract

For renewable energy technology to become ubiquitous, it is imperative to develop efficient oxygen evolution reaction (OER) electrocatalysts, which is challenging due to the kinetically and thermodynamically unfavorable OER mechanism. Transition metal carbides (TMCs) have recently been investigated as desirable OER pre-catalysts, but the ability to tune electrocatalytic performance of bimetallic catalysts and understand their transformation under electrochemical oxidation requires further study. In an effort to understand the tunable TMC material properties for enhancing electrocatalytic activity, we synthesized bimetallic FeCo nanocarbides with a complex mixture of FeCo carbide crystal phases. The synthesized FeCo nanocarbides were tuned by percent proportion Fe (*i.e.* % Fe), and analysis revealed a non-linear dependence of OER electrocatalytic activity on % Fe, with a minimum overpotential of 0.42 V (15–20% Fe) in alkaline conditions. In an effort to understand the effects of Fe composition on electrocatalytic performance of FeCo nanocarbides, we assessed the structural phase and electronic state of the carbides. Although we did not identify a single activity descriptor for tuning activity for FeCo nanocarbides, we found that surface reconstruction of the carbide surface to oxide during water oxidation plays a pivotal role in defining electrocatalytic activity over time. We observed that a rapid increase of the (Fe_*x*_Co_1−*x*_)_2_O_4_ phase on the carbide surface correlated with lower electrocatalytic activity (*i.e.* higher overpotential). We have demonstrated that the electrochemical performance of carbides under harsh alkaline conditions has the potential to be fine-tuned *via* Fe incorporation and with control, or suppression, of the growth of the oxide phase.

## Abstract 2

Fe-content in FeCo nanocarbides tunes electrocatalytic activity towards the oxygen evolution reaction, and formation of crystalline oxide occurs during electrocatalysis.

## Graphical abstract

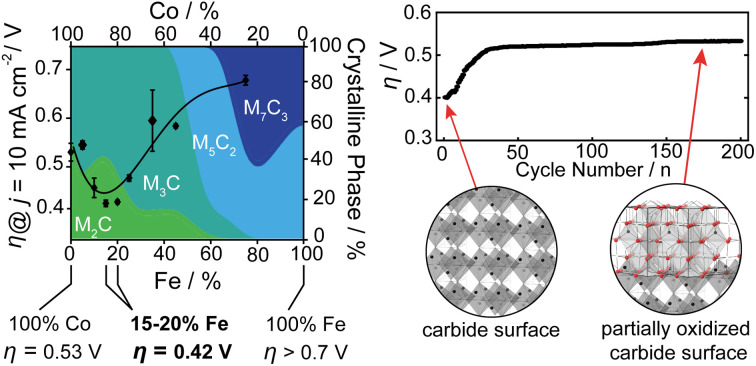

## Introduction

Electrochemical water splitting offers a promising route for sourcing green hydrogen, a renewable energy alternative to fossil fuels.^1–3^ However, the anodic four-electron oxygen evolution reaction (OER) mechanism is kinetically sluggish and thermodynamically unfavorable under alkaline conditions.^4,5^ Despite tremendous efforts in the search for new catalysts to utilize in electrochemical water splitting systems,^6,7^ costly ruthenium and iridium oxide (RuO_2_ and IrO_2_) electrocatalysts persist as the only viable options for industrial implementation.^8–11^ Therefore, the development of alternative highly efficient, earth-abundant and low cost electrocatalysts for the OER remains crucial.

Nanoparticle electrocatalysts have attracted considerable interest for the replacement of bulk noble metal oxide catalysts due to their increased surface area to volume ratio, exposure of more active sites to reduce the amount of material needed to undergo reactions like the OER,^12–14^ and offering a high degree of physical and chemical property tunability for the modulation of catalytic performance.^15,16^ In particular, earth-abundant transition metal (TM)-based nanocatalysts have been reported to have comparable electrochemical performance to noble metal-based catalysts.^13^ Recent studies have investigated transition metal carbides (TMCs) as low-cost alternative electrocatalysts for the hydrogen evolution reaction (HER) in acidic conditions,^17–19^ however, few reports have discussed their use for catalyzing the OER in alkaline conditions.^20–22^ TMCs possess high electrical conductivity, high chemical stability, and are resistant to corrosion at both the bulk and surface levels, all properties that are advantageous for water splitting electrocatalysts.^23,24^ While TMCs often do not have competitive OER activity and kinetics in comparison to high-performance TM-based oxides and layered double hydroxide (LDH) materials,^25,26^ carbon materials often have more potential for scalable, low-cost fabrication processes and high thermal stability and remarkable conductivity for potential use in water electrolyzer applications.^18,27^ Therefore, developing an in-depth understanding of these materials can aid in rational design of TMC materials with high OER electrocatalytic efficiency.

We recently investigated the electrocatalytic OER performance of monometallic TM-nanocarbides, finding that Co > Ni > Fe for both electrocatalytic activity and stability with Co being our best performer, while the oxide thickness layer for post-catalytic OER nanocarbides decreased in the order Fe > Co > Ni.^22^ There have been efforts towards designing enhanced electronic properties of TMCs for OER catalysis.^20,28^ In particular, the incorporation of Fe with another metal in a bimetallic system for improved electrocatalytic activity has been widely studied, and these materials often outperform monometallic catalysts as a result of synergistic effects due to various structure and composition-dependent enhancement in active sites.^28–35^ In particular, studies claim that mixed crystalline phases and increased disorder that often result from multi-metal incorporation have been shown to modify local electronic structures, leading to enhancement in activity towards the OER.^33,36,37^ These synergistic effects are not well understood for bimetallic carbide systems and could be potential activity predictors for designing future bimetallic carbide catalysts.

Non-oxide-based catalysts are often known for undergoing surface termination changes *via in situ* electrochemical oxidation, to produce thin oxide/hydroxide surface layers that are known for enhancing electrocatalytic activity.^21,38–41^ However, our previous study on monometallic TMCs revealed that the thickest oxide layer formed on the Fe carbide (as compared to Co and Ni), but this material exhibited the lowest electrocatalytic activity and poor stability.^22^ We are therefore motivated to understand how to tune electrocatalytic activity in bimetallic FeCo carbides, given that we previously observed that the monometallic Co carbide was the highest performing electrocatalyst, and to explore the phenomenon of oxide layer transformation on carbide surfaces.

In this study, Fe_*x*_Co_1−*x*_C_*y*_ nanocarbides were synthesized from a single-source Prussian blue analogue (PBA) precursor, using a previously established method, which offers a potential route to economical bimetallic carbides for use as industrial OER electrocatalysts. The percent proportion of Fe (to Fe and Co) was changed, herein referred to as % Fe (*i.e. x* × 100%), for a series of bimetallic Fe_*x*_Co_1−*x*_C_*y*_ nanoparticles, which resulted in various crystal phases across the entire composition range. These bimetallic carbides, and the monometallic Fe and Co carbides, were analyzed to reveal that optimal OER electrocatalytic activity was achieved for the samples that were synthesized to contain 15–20% Fe with a geometric normalized overpotential of 0.4 V. Our results suggest that Fe content is not the sole contributor for tunability of electrocatalytic activity, rather it works in synergy with resulting structural and oxide surface layer composition changes of the Fe_*x*_Co_1−*x*_C_*y*_ nanocarbides.

## Experimental

### Materials

All commercially available reagents were used without further purification. Precursors for FeCo PBAs were K_3_Co(CN)_6_ and K_3_Fe(CN)_6_ (Sigma Aldrich, >99%), KCl (Sigma Aldrich, 98%), CoCl_2_·6H_2_O (Thermo Fisher, >99%), and FeCl_2_·4H_2_O (Thermo Fisher, >99%). Solvents used for synthesis were ultrapure water (18.2 Ω cm^−1^ at 25.0 °C, Thermo Scientific Barnstead E-Pure ultrapure water purification system), octadecylamine (ODA) (Thermo Fisher, 90%), acetone (VWR, ACS Grade) and toluene (VWR, ACS Grade).

### Synthesis of FeCo Prussian blue analogue (PBA) precursors

Two precursor solutions were prepared, and upon combination a precipitation reaction occurred to form the PBA. Briefly, *x* mmol K_3_Fe(CN)_6_ and 1 − *x* mmol K_3_Co(CN)_6_ (where *x* = 0, 0.1, 0.3, 0.5, 0.7, 0.9, 1), and 5 mmol of KCl in 100 mL of ultrapure water, comprised solution 1. Solution 2 comprised 1 mmol of either FeCl_2_ (to make PBAs of >50% Fe) or CoCl_2_ (to make PBAs of <50% Fe) in 200 mL of ultrapure water. Solution 2 was added dropwise to solution 1 at a rate of 5 mL min^−1^ and vigorously stirred. The subsequent reaction solutions were left for 18 h while stirring to grow the PBAs. The PBAs were collected *via* centrifugation, washed with 300 mL of ultrapure water and dried on the benchtop at room temperature. The PBA precursors were characterized using scanning electron microscopy ((SEM), Fig. S1†), powder X-ray diffraction ((pXRD), Fig. S2†), and X-ray fluorescence ((XRF), Table S1†).

### Synthesis of FeCo nanocarbides

200 mg of solid PBA and 40 mL of ODA were heated to 330 °C, under inert atmosphere for 24 h. After 24 h, the reaction was quenched using toluene and the resultant nanocarbide was collected *via* magnetic separation. The nanoparticles were washed with toluene (3×), acetone (1×), ultrapure water (3×), and again with acetone (1×), then dried in an oven at 100 °C for 15 minutes. The nanoparticles were structurally characterized using pXRD (Rigaku Miniflex benchtop powder diffractometer, Cu Kα (ESI Fig. S3 and S4†)). Elemental composition was confirmed using XRF spectroscopy (Panalytical Epsilon X-ray fluorescence analyzer, ESI Table S1†). Morphology and size analyses were executed using transmission electron microscopy (TEM, FEI CM300 FEG).

### Synthesis of FeCo oxides

200 mg of solid PBA was loaded into an aluminum boat and placed into a Lindberg tube furnace. The PBA was subsequently heated to 300 °C with a ramp rate of 60 °C min^−1^, for 30 minutes. The resultant oxides were structurally characterized with pXRD (ESI Fig. S5 and S6†).

### Materials characterization

pXRD patterns of PBAs, PBA derived carbides and PBA derived oxides were collected at room temperature on a Rigaku Miniflex powder diffractometer (Cu Kα source, *λ* = 1.54 Å, ESI Fig. S2–S4†). The contributions of various crystalline phases were fitted and calculated as a percentage for each Fe_*x*_Co_1−*x*_C_*y*_, using fits shown in Fig. S4.† pXRD measurements on post-OER samples were performed on a Rigaku Synergy single crystal diffractometer running in powder diffraction mode (Mo Kα source, *λ* = 0.71 Å). The bimetallic ratios in both PBA and nanocarbide were confirmed using XRF on a Panalytical Epsilon XRF analyzer (Cu Kα source, ESI Table S1†). X-ray photoelectron spectroscopy (XPS) was performed on as-synthesized powders deposited on carbon tape using a PHI 5100 X-ray photoelectron spectrometer (Mg Kα source) with a pass energy of 22.36 eV. The XPS spectra were fitted using CasaXPS software. Samples were Ar^+^-sputtered using a sputtering gun at 5 keV and 1 μA for 15 minutes to reveal underlying carbide features. All samples were calibrated to the aliphatic carbon assignment (C 1s, 284.8 eV). Size and morphology of PBA precursors were investigated *via* SEM imaging (FEI Nova 400, 15 keV, Fig. S1†). Size, size dispersity, and morphology of the nanocarbides were estimated using ImageJ software (sample size = 100 particles) *via* TEM images, collected on a Tecnai Osiris TEM operating at 200 kV.

### Electrode preparation

A catalyst ink suspension was prepared using catalyst powder (1.3 mg, 2 mL total volume) in a solution mixture of 10% Nafion (5% (w/w) in water/1-propanol, Beantown Chemical), 6% ethanol, and 84% deionized water. The mixture was then sonicated for 5 min, until a homogeneous black ink formed. Catalyst ink (31 μL) was drop casted onto the surface of a 5 mm diameter glassy carbon (GC) rotating disk electrode (RDE) (Pine Research Instrumentation) with a nanoparticle mass loading of 0.1 mg cm^−2^. The samples were dried for 1–2 h in air at room temperature to achieve a uniform thin film (shown in the SEM image in Fig. S7†).

### Electrochemical measurements

All electrochemical measurements were performed using a RDE setup equipped with an electrode rotator (WaveVortex 10, Pine Research Instrumentation) set to 1500 rpm, connected to a potentiostat (model CH 660E, CH instruments) within a compartmentalized electrochemical glass cell filled with approximately 250 mL of 1.0 M KOH. A three-electrode setup was used with a GC RDE as the working electrode, a Ag/AgCl reference electrode (1.0 M KCl internal filling solution), and a graphite rod counter electrode.

The electrochemical surface area (ECSA) was determined for each sample using the double layer capacitance, *C*_dl_, measured by cyclic voltammetry (CV), so that current densities could be estimated (example shown in ESI Fig. S8†).^42,43^ The charging current, *i*_c_, is proportional to the potential scan rate, *v*, shown in the relationship

1 *i*_c_ = *vC*_dl_

By varying the scan rate (10, 20, 50 and 100 mV s^−1^), a plot of *i*_c_ as a function of *v* will yield a straight line where *C*_dl_ is the gradient, using CVs recorded in a designated potential window of the non-faradaic region of the CV, shown in example CV in Fig. S8† as 0.81 to 1.01 V *vs.* reversible hydrogen electrode (RHE). ECSA was calculated using the determined value of *C*_dl_ using

2 ECSA = *C*_dl_/*C*_s_

where *C*_s_ is the specific capacitance of the material. We used a value for *C*_s_ of 45 μF cm^−2^ for the Fe_*x*_Co_1−*x*_C_*y*_ samples, based on reported values in literature for TMs on GC electrodes in the range of 30–70 μF cm^−2^.^44,45^

In 1.0 M KOH (pH = 13.8) electrolyte, the potentials against Ag/AgCl can be converted to potentials *vs.* the reversible hydrogen electrode (RHE) at 25 °C using

3 *E*_*vs.* RHE_ = *E*_*vs.* Ag/AgCl_ + 0.059 pH

which was used to calculate the overpotential, *η*, using

4 *η* = *E*_*vs.* RHE_ − 1.23 V

Additionally, a master reference electrode (not used in experiments) was compared against the Ag/AgCl reference electrode used experimentally, and was observed to change no more than a 5 mV to ensure a stable, well-defined electrochemical potential.

While the OER linear sweep voltammograms shown in the manuscript were not corrected for *iR* compensation, curves that were *iR*-corrected are included in the ESI (Fig. S9†), which eliminate contributing resistance factors. The potential was corrected using

5 *E*_*iR*-corrected_ = *E*_*vs.* RHE_ − *iR*_u_

where *i* is current, and *R*_u_ is the uncompensated resistance determined by electrochemical impedance spectroscopy (EIS) measurements at a potential of 0.6 V *vs.* Ag/AgCl.

Tafel slopes were calculated from the linear kinetic region of the Tafel plot, *i.e.* log(current density) *vs.* overpotential, at the early onset current in the LSV curves. Electrochemical stability measurements were performed for 200 repetitive CV cycles, with a potential range of 1.0 to 1.8 V *vs.* RHE, using a scan rate of 5 mV s^−1^. For the preparation of samples analyzed by pXRD post-OER, nanomaterial was drop casted onto a GC wafer electrode (glassy carbon plate, 2 mm thick, Thermo Fisher) setup with an estimated mass loading of 0.8 mg cm^−2^.

## Results and discussion

### Electrocatalytic activity of Fe_*x*_Co_1−*x*_C_*y*_ exhibits non-linear dependence on % Fe

Here, PBA derived FeCo nanocarbides were rationally designed using previously established synthetic conditions, and Fe and Co proportions were finely tuned for controlling OER activity. XRF was used to determine the elemental composition, and the ratio of Fe and Co was maintained from PBA precursor to carbide (ESI Table S1†). There was reasonable agreement between the measured Fe : Co ratio, and desired ratio based on synthesis, so all samples are referred to by the desired % Fe throughout this work. The electrocatalytic activity and stability of the nanocarbides towards the OER was evaluated in 1.0 M KOH, using a three-electrode set up and a mass loading of 0.1 mg cm^−2^. Electrocatalytic activities of the FeCo nanocarbides were evaluated by extracting the overpotential required to achieve a current density of 10 mA cm^−2^ from linear sweep voltammograms (LSVs). This value is the benchmarking standard for current density expected at the anode for an artificial photo-synthetic device yielding 10% efficiency at 1 sun illumination, and serves as a useful comparison for our samples and literature.^43,46^ The electrochemically active surface areas (ECSAs) were determined from the electrochemical double layer capacitance of the drop casted surface, to allow for comparison of intrinsic activity between samples (representative example shown in Fig. S9†). This was necessary because the materials have both crystalline and amorphous features (confirmed by XPS in Fig. 4), the latter of which tend to have enhanced ECSAs.

**Fig. 1:**
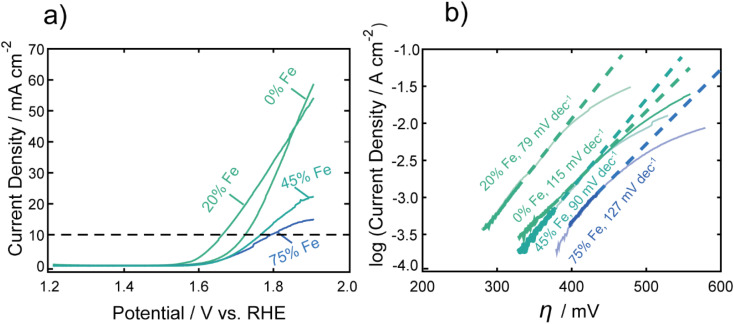
(a) Representative linear sweep voltammograms of FeCo nanocarbides in 1.0 M KOH, with a dashed line denoting the benchmarking standard current density of 10 mA cm^−2^. Note that RuO_2_ achieved an overpotential of 0.36 V at 10 mA cm^−2^ (per geometric surface area). (b) The linear regions of the Tafel plots were fitted, using the kinetically-controlled region of the voltammetry from part (a) to determine Tafel slopes, indicated by the dashed lines. Note that the Tafel slope obtained for RuO_2_ was 85 mV dec^−1^.

Fig. 1a shows representative LSVs of the nanocarbides, with their corresponding Tafel slopes in Fig. 1b. FeCo nanocarbides containing 20% Fe exhibited lower overpotentials and steeper voltammetric slopes than those below or above this % Fe (Fig. 1a). Also, the more active OER carbide electrocatalysts, between 0 and 20% Fe, exhibited an exponential increase of current density as potential increased, which is to be expected based on the Butler–Volmer equation. However, the voltammetry in Fig. 1a also shows that as % Fe increased above 20%, the rate of increase of current density was suppressed at the highest potentials and higher overpotentials (lower activity) at 10 mA cm^−2^ were observed. It is important to mention that these catalysts have a low mass loading (0.1 mg cm^−2^) compared to significantly higher loadings used in other catalyst studies, and it is well known that an increase in catalyst loading can be utilized to enhance electrocatalytic reaction rates. The voltammetry for carbide catalysts with higher % Fe exhibiting more diffusion-controlled behavior could suggest that the accessibility of the catalyst towards the electrolyte solution is hindered for nanocarbide compositions with higher Fe content, and could require higher mass loadings to overcome diffusion effects.^47^ Attempts to correct electrocatalytic voltammograms with mixed kinetic and mass transport control on a macroelectrode have been implemented using computational studies, but are time-intensive to implement.^48^

To consider solution resistance factors, *iR* drop compensation was performed on CoC and FeCoC (15% Fe), shown in ESI Fig. S9.† We found that the overpotentials extracted at a current density of 10 mA cm^−2^ yielded an inappreciable shift in overpotential of ∼13 mV when *iR* corrected. These voltammetric differences warranted Tafel analysis to gain insight into the kinetics of the electrocatalytic OER reaction, shown in Fig. 1b. The linear region of the Tafel plot was fitted from the kinetically controlled region of the voltammogram to provide Tafel slopes of nanocarbides with varying % Fe. These fitted slopes are shown as dashed lines in Fig. 1b, which do not fit the portions of the voltammograms in which diffusion effects become significant. We can assume based on the lower Tafel slopes shown for nanocarbides with 45 and 75% Fe (90–127 mV dec^−1^) in Fig. 1b that these are poor performing catalysts as compared to nanocarbides with 20% Fe, without additional analysis.

The monometallic Co nanocarbide (*i.e.* 0% Fe) achieved an overpotential of 0.53 V (at 10 mA cm^−2^), and the 100% Fe carbide was unable achieve a current density of 10 mA cm^−2^ in this potential window (shown in Fig. S10†). The best performing FeCo nanocarbide electrocatalysts, *i.e.* 15–20% Fe, yielded a lower, enhanced overpotential of 0.40 V (geometric corrected) and 0.42 V (ECSA corrected) compared to the monometallic Fe and Co carbides. For comparison, an industrial electrocatalyst RuO_2_ was tested under the same electrochemical conditions and mass loading, which gave an overpotential of 0.36 V at 10 mA cm^−2^, comparable to other RuO_2_ values shown in literature (0.38 V).^12^ Higher intrinsic activities are often predicted for catalysts with higher mass loadings. Electrocatalytic activity measurements were performed with higher mass loadings of 0.8 and 0.4 mg cm^−2^ using one of our best performing FeCo nanocarbides with 15% Fe. Samples prepared with higher mass loadings resulted in a higher rate of OER current and slightly lower overpotentials, with the lowest geometric overpotential achieved with a mass loading of 0.8 mg cm^−2^ (0.38 ± 0.01 V) and current densities of ∼105 mA cm^−2^ (Fig. S11†). Mechanical instabilities such as the thin nanomaterial film peeling from the surface and sample flaking off the electrode surface occurred in long-term electrochemical stability interrogation of samples with higher mass loading (>0.8 mg cm^−2^), which motivated us to choose a lower mass loading of 0.1 mg cm^−2^ for this study.

**Fig. 2:**
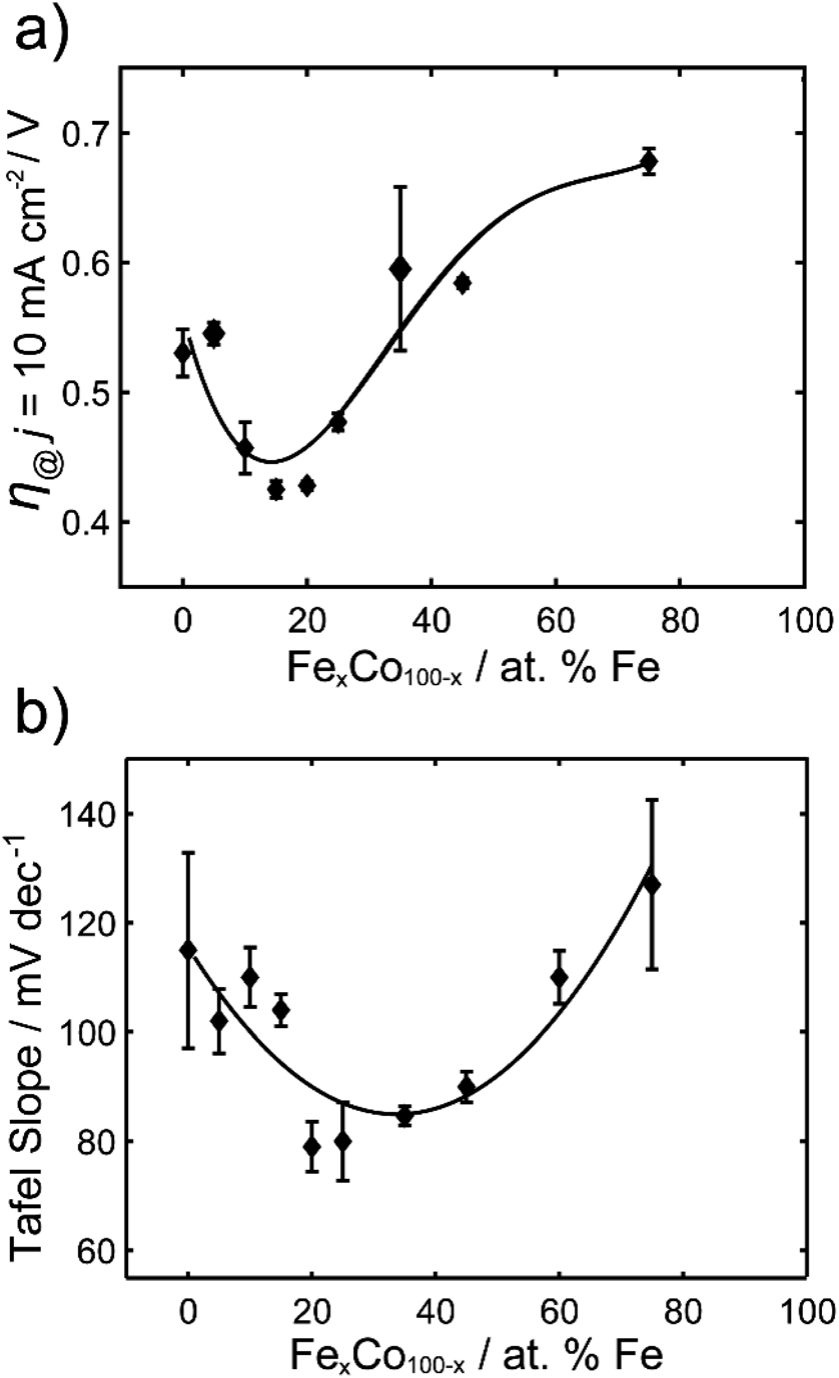
(a) Overpotentials (*n* = 3) required to achieve 10 mA cm^−2^ (per ECSA) for Fe_*x*_Co_1−*x*_C_*y*_ of varying % Fe, in 1.0 M KOH. (b) Tafel slopes for Fe_*x*_Co_1−*x*_C_*y*_ of varying % Fe.

The overpotentials at 10 mA cm^−2^ were extracted from each voltammogram and are plotted against the % Fe in Fig. 2a. A U-shaped curve (polynomial fit to guide the reader) is observed with a minimum overpotential between 15–20% Fe. In Fig. 2b, the corresponding Tafel slopes showed a similar U-shaped curve, with a favorable minimum Tafel slope observed between 20–25% Fe. Tafel plots allow for the kinetic region of a voltammogram to be analyzed, although unlike for the HER, the value of the Tafel slope cannot be used for directly predicting the mechanism of the OER, given the multi-electron reaction and many possible intermediates.^4,49,50^ When the reaction rate is limited by the charge transfer reaction, Tafel slopes of 120 mV dec^−1^, 90 mV dec^−1^, 60 mV dec^−1^, and 30 mV dec^−1^ can be correlated to 1, 2, 3, and 4 electron transfer processes,^51^ respectively, under alkaline conditions. Comparison of Tafel slopes, albeit without full interpretation, is useful to compare the relative kinetics of the various samples. The most favorable kinetics for the OER were observed at 20–25% Fe, with a Tafel slope of 79 mV dec^−1^, comparable to a Tafel slope of 85 mV dec^−1^ for RuO_2_ and suggesting a 2 electron-transfer rate determining step. FeCo nanocarbides with lower Fe content, *i.e.* 0–15% Fe, have Tafel slopes ranging from approximately 115 to 104 mV dec^−1^, respectively, suggesting that the 0% Fe sample is closest to the 1 electron-transfer rate determining step. Similarly, 75% Fe has a high Tafel slope of 127 mV dec^−1^ that corresponds to a 1 electron-transfer rate determining step, suggesting that <15% Fe and >75% Fe both have less favorable electron transfer kinetics.

The results from the FeCo nanocarbide system reveal optimal geometric and ECSA normalized overpotentials (for 15–20% Fe) of 0.40 V and 0.42 V, respectively, which are competitive to a geometric-normalized overpotential of a Co_2_C OER pre-catalyst reported by Mullins and coworkers of 0.46 V.^21^ When comparing electrocatalysts in literature, it is important to note that there are various methods by which the materials are attached to a substrate electrode. Electrode modification methods other than drop casting, such as electrodeposition and sputtering, will result in different film thicknesses and catalytic loading, which can influence the measured overpotentials. While our study is not motivated in simply lowering this benchmarking overpotential, and is more concerned with understanding which (and how) material properties dictate the overpotential for carbides, it is nonetheless important to consider where the carbides lie in relation to the state-of-the-art and other competitive catalysts. Our best FeCo nanocarbide (for 15% Fe) had an overpotential of 0.38 V (*j* = 10 mA cm^−2^) at a mass loading of 0.8 mg cm^−2^, which is competitive to a geometric-normalized overpotential of a Co_2_C OER pre-catalyst reported by Mullins and coworkers of 0.46 V.^21^ Other examples in literature such as FeCo phosphide has an overpotential of 0.37 V (for 50% Fe),^52^ and FeCo(OOH) has an overpotential of 0.35 V (60% Fe), 31 which are comparable to our system. The lowest overpotentials in the field have been demonstrated for FeCo-layered double hydroxide (LDH) nanosheets, with an overpotential of 0.28 V,^53^ and FeCo-oxyhydroxide (OOH) nanosheet, with an overpotential of 0.21 V.^54^ For comparison with our FeCo nanocarbides, more examples of reported overpotentials are provided in the ESI (Table S2†).

Fe's role in regulating OER activity for multimetallic systems has been suggested to result from: the favorable binding energies of intermediate species in the OER inducing stabilization of the crystal lattice,^32^ Fe^3+^ acts as the catalytic active site in both FeCo and FeNi materials,^31,33^ Fe has increased conductivity over other TMs,^31^ and the regulation of charge transfer energies in a mixture of Co^4+^ and Fe^4+^ ions.^55^ In this work, we attempted to identify the source of electrocatalytic enhancement that occurs when combining Fe and Co in the bimetallic carbide catalysts, exploring the role of key activity descriptors. In the following section, we have investigated the effects of tuning material composition and structure, such as crystal phase composition and surface chemical states, on regulating electrocatalytic activity.

### Effects of material composition and structural properties on OER electrocatalytic activity

**Fig. 3:**
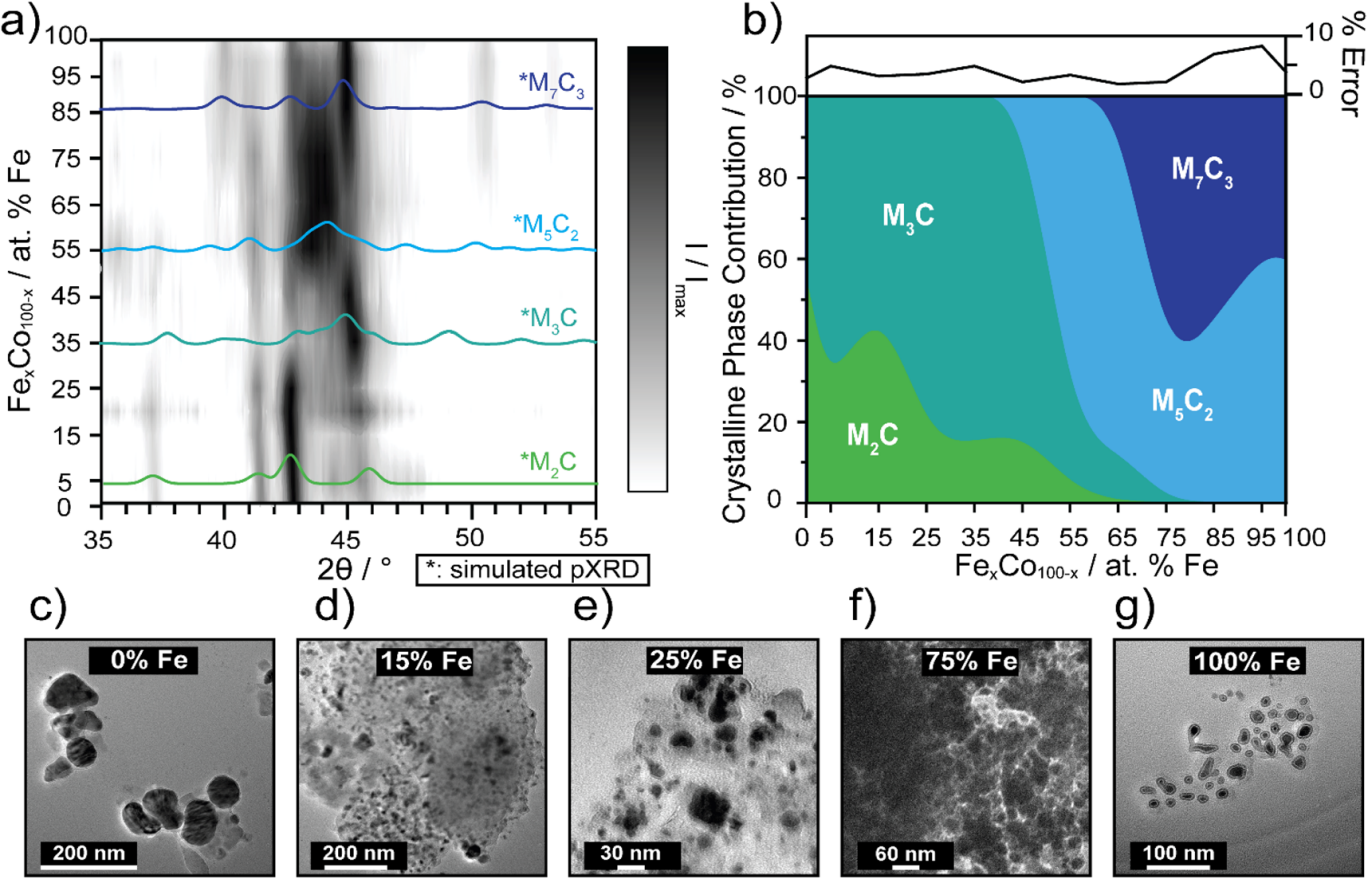
(a) 3D contour plot tracking the evolution of the major carbide phase as a function of % Fe, using pXRD patterns. The black intensity represents the XRD signal, *I*, normalized to the maximum signal, *I*_max_, where the most intense peaks appear darkest. Phase references are broadened to reflect 10 nm materials and overlaid (in color) to highlight differences. The references shown are for M_7_C_3_ (dark blue, ICSD: 76830), M_5_C_2_ (light blue, ICSD: 423885), M_3_C (blue-green, ICSD: 43521) and M_2_C (green, COD: 1528415). (b) Proposed phase diagram of metastable bimetallic carbides, where relative phase contributions are plotted against % Fe. The error plot (top) represents the % error (±) in each fit. Fits for select samples are shown in ESI Fig. S4.† TEM images of select Fe_*x*_Co_1−*x*_C_*y*_ for (c) 0% Fe, (d) 15% Fe, (e) 25% Fe, (f) 75% Fe, and (g) 100% Fe. Sizes of the nanocrystals increase, as the Fe content decreases, based on histogram size analysis (*n* = 50–100).

Carbides are known to have amorphous and graphitic-type carbon that could influence phase,^56^ therefore impacting electrocatalytic activity as the carbide crystallinity and crystal structure is tuned.^57^ The preparation of pure-phase Fe carbide materials is notoriously difficult to achieve under mild synthesis conditions, often resulting in mixed phase materials.^19,20,56,58^ Strain in materials can often be the result of substitutional doping and disorder.^59–61^ To investigate whether crystal phase composition of the nanocarbides plays a role in the electrochemical activity, pXRD phase analysis was used to reveal an evolution of crystal phases across the various percentages of Fe (Fig. 3a and b).

The simulated pXRD patterns of the four identified phases are shown in Fig. 3a, which are overlaid on a contour plot of 2*θ vs.* Fe percentage, in which the black intensity is normalized to most prominent peak in the pXRD pattern. Fig. 3b represents approximate individual phase contributions, of the four unique phases identified in the Fe_*x*_Co_1−*x*_C_*y*_ system, across the range of samples. However, identifying the amount of each phase present was a non-trivial task, due to diffraction pattern overlap and differences in diffraction intensity. To deconvolute contributions of each phase towards the overall diffraction patterns, whole pattern fitting was executed using Rigaku SmartLab Studio II software (Fig. S4†). According to the fits, all samples are mixed phase, containing a minimum of two crystal phases. From 0–45% Fe, the carbides have approximately 60–85% M_3_C (ICSD: 43521, hexagonal) phase and 15–40% M_2_C (COD: 1528415, orthorhombic) phase. From 55–65% Fe, the M_5_C_2_ persists as the major phase and decreases in abundance at 65% Fe, where the final phase M_7_C_3_ (ICSD: 76830) evolves in and is mixed with M_5_C_2_, up to 100% Fe. There are key differences in electrocatalytic activity observed between different mixed-phase regions shown in Fig. 1 and 2, such as 75% Fe (mixed M_5_C_2_/M_7_C_3_) and 0% Fe (mixed M_2_C/M_3_C), which exhibit an overpotential difference of 150 mV. Although phase may effect catalysis in conjunction with tuning Fe composition, this is not the only activity descriptor for the FeCo carbide catalysts. This is evidenced by nanocarbides between 0–20% Fe which have similar phase compositions, but differing overpotentials (Fig. 2b).

Another factor considered for optimizing electrocatalytic activity was the size of the nanocrystal, however our results showed that the size of the carbide nanoparticles could not be correlated to electrocatalytic activity. In agreement with previous studies,^22,62^ the resultant carbide size was proportional to the size of the mesocrystal precursor (SEM images shown in Fig. S1†). The size and morphology of the nanocarbide particles were analyzed using TEM analysis (Fig. 3c–g). However, due to the presence of significant amounts of amorphous carbon surrounding our nanocarbides, the images were only used to estimate particle size and shape. The monometallic Co (*i.e.* 0% Fe) carbide particles were significantly larger than the other bimetallic carbide nanocrystals, as shown in the TEM images in Fig. 3 for 0% Fe (51 ± 6 nm), 15% Fe (9 ± 6 nm), 25% Fe (9 ± 3 nm), 75% Fe (14 ± 3 nm), and 100% Fe (8 ± 2 nm). Based on TEM images shown in Fig. 3c–g, and in our previous study, the resulting carbide nanocrystals are highly disordered, with stacking faults likely present.^22^ Although stacking faults^63^ and NP size^16^ are often linked to tuning electrocatalytic activity, our results show that the minimum overpotential of 0.42 V achieved cannot be attributed to differences in particle size or morphology, as particles containing 15–100% Fe the have similar nanocrystal sizes, yet the electrocatalytic activity changes significantly.

**Fig. 4:**
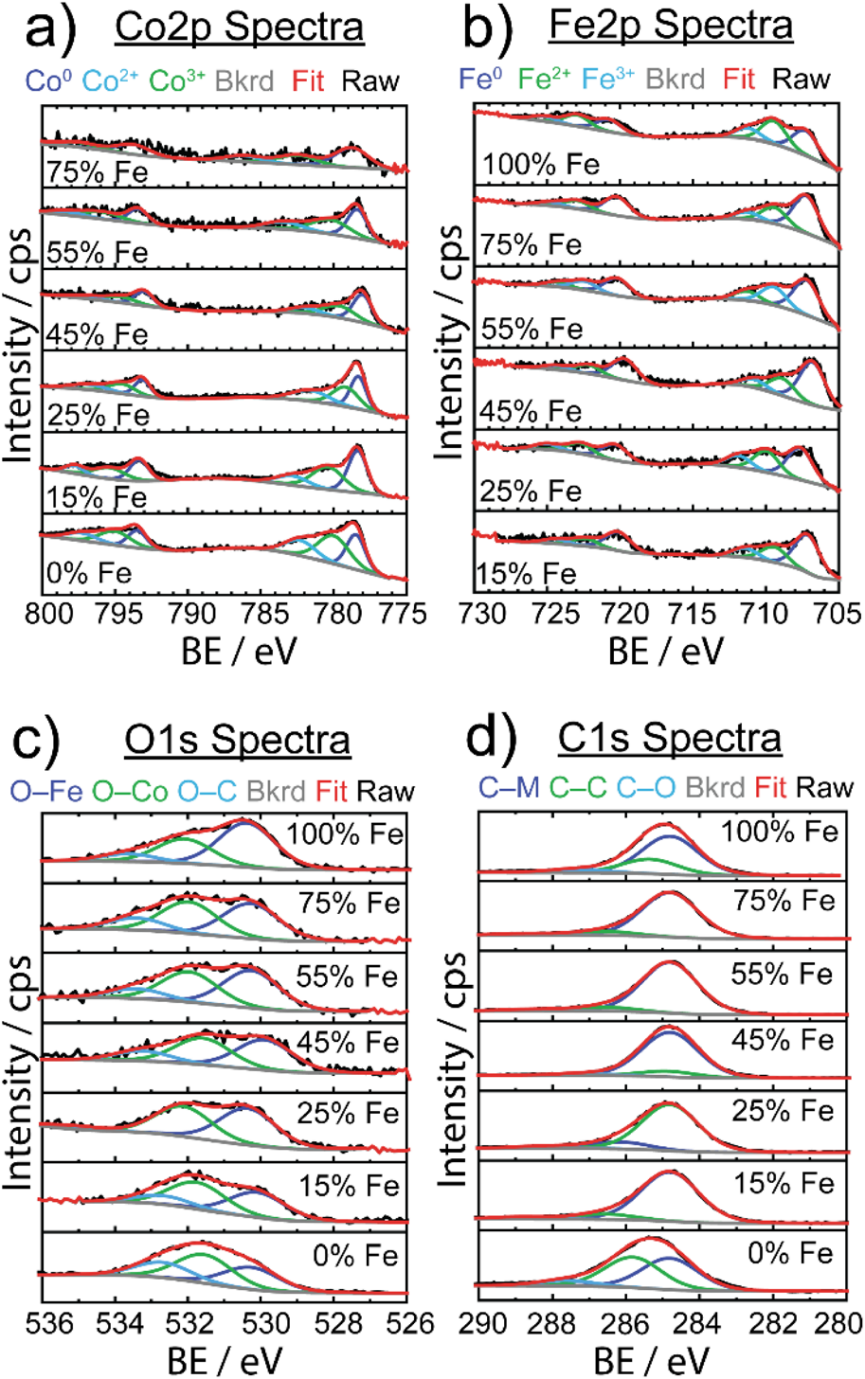
Stacked X-ray photoelectron spectra for Fe_*x*_Co_1−*x*_C_*y*_ samples of varying Fe concentrations are shown for (a) Co 2p, (b) Fe 2p, (c) O 1s, and (d) C 1s with respective contributions of chemical species below. Black lines represent raw data, red lines represent overall fits, the gray lines show the background (bkrd) and specified chemical species are shown in dark blue, green and light blue lines.

XPS was used to investigate the surface structure of the as-synthesized Fe_*x*_Co_1−*x*_C_*y*_ materials with varying % Fe (Fig. 4), with chemical shifts obtained from fits in ESI Table S3.† For the Co 2p spectra in Fig. 4a, three Co species were identified: Co^0^ (778 eV 2p_3/2_), Co^2+^ (780–786 eV 2p_3/2_), and Co^3+^ (779–785 eV 2p_3/2_), indicative of the carbide and mixed valence oxide surface species, respectively.^64^ The observed Co^2+^ and Co^3+^ species observed in as-synthesized samples are consistent with finding from Mullins and coworkers,^21^ suggesting the presence of a thin-layer amorphous oxide on the surface. In particular, the evidence of a broadened 2p_3/2_ peak at ∼795 eV is indicative of the presence of a spinel Co_3_O_4_ compound, which is in agreement with our result from post-OER pXRD analysis (*vide infra*). The Fe 2p spectra revealed three Fe species: Fe^0^ (707 eV 2p_3/2_) resulting from metal carbide, and Fe^2+^ (709 eV 2p_3/2_) and Fe^3+^ (711 eV 2p_3/2_) resulting from a mixed valence oxide at the surface. The C 1s spectra revealed three unique species: metal to carbon bonding (M–C, 284.8 eV) present in the carbide material, carbon to carbon bonding (C–C, 286 eV) from ligand, carbide, and carbon tape support, and carbon to oxygen bonding (C–O, 288 eV) attributed to ligand and oxygen passivation *via* ambient conditions.

In the O 1s spectra, three species were identified, and attributed to oxygen bonding to each of Fe and Co at the surface (O–Fe: 531–532 eV, O–Co: 530 eV), and oxygen to carbon bonding (O–C, 533 eV) due to ligand and amorphous surface oxide contributions. Although there was some variability in chemical shifts in the Co 2p and Fe 2p spectra, there were no detectable changes in electronic structure to explain electrocatalytic enhancement.

A study on Fe-doped molybdenum carbide catalysts noted that although there were appreciable differences in the electrocatalytic activity of pure and Fe-doped molybdenum carbide, there was no significant difference in oxidation state shifts upon addition of Fe, similar to our data (Fig. 4).^65^ However, to better understand the thin amorphous oxide layer detected on the carbide samples, we implemented the difference in oxide before and after Ar^+^ ion etching to quantify the amount of surface oxide (shown in Fig. S12†).

Interestingly, 15% Fe had the smallest change in oxide amount after sputtering, which could suggest a more stable, or possibly thinner, oxide surface in comparison to the other samples. In our previous studies on monometallic TMCs, it was observed that higher electrocatalytic activity was correlated to a thinner oxide surface layer.^22^ Although we did not identify a key activity descriptor to explain the role of Fe composition in tuning OER activity, we are motivated to better understand the role of surface oxide for regulating electrocatalytic activity.

### Electrochemical transformation of Fe_*x*_Co_1−*x*_C_*y*_ during oxygen evolution reaction under alkaline conditions

**Fig. 5:**
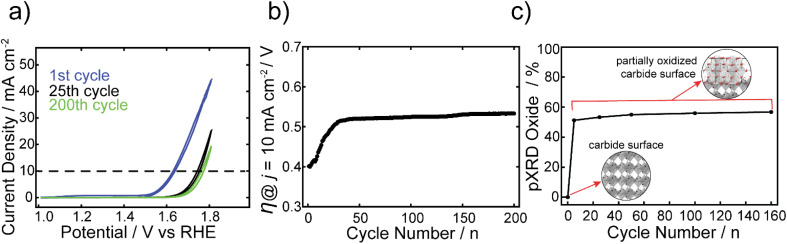
(a) CVs of the 1st, 25th, and 200th cycles at a scan rate of 5 mV s^−1^ for the FeCo nanocarbide, containing 15% Fe. (b) Overpotentials were extracted from CVs at a current density of 10 mA cm^−2^ over 200 cycles. (c) Plot showing phase contributions from (Fe_0.5_Co_0.5_)_2_O_4_ (*i.e.* oxide) derived from XRD analysis of 15% FeCo post-electrocatalytic OER, with increasing CV cycles, using a Mo Kα source. Insets shown in (c) depict a carbide surface with an M_2_C crystalline phase (orthorhombic lattice) and a partially oxidized carbide surface containing (Fe_0.5_Co_0.5_)_2_O_4_ (cubic lattice), embedded in the surface layer. These lattice structures were generated using CrystalMaker software.

The electrocatalytic stability of one of the best performing FeCo nanocarbides, 15% Fe, was tested and compared against commercial RuO_2_ nanoparticles. The Fe_*x*_Co_1−*x*_C_*y*_ samples were tested using CV repetitive cycling (Fig. 5a), so that overpotentials could be extracted at 10 mA cm^−2^ from each voltammogram (Fig. 5b), in alkaline conditions using a RDE setup. It was evident that although the initial OER activity was greater for commercial RuO_2_ than the nanocarbides in the first cycle, the electrocatalytic OER stability of the RuO_2_ nanoparticles was greatly affected by harsh OER alkaline conditions upon further cycling (ESI Fig. S11†). Given the rapid loss in activity, after ten cycles the current density no longer achieved the benchmarking current density of 10 mA cm^−2^. Therefore, the maximum current density observed at 1.8 V was extracted from the CVs to show the loss of performance. The current density decreased by more than half after just ten cycles, and by 92% of the original value after 100 cycles (Fig. S13†).

In our previous work, we found that the Co carbide resulted in robust electrochemical stability with <2% increase of overpotential over 100 CV cycles.^22^ In contrast, the 15% Fe nanocarbide from this study showed a ∼110 mV increase in overpotential (loss of activity) in the first 30 cycles (Fig. 5). Between 30 and 200 cycles the overpotential remained relatively stable, with <4% change in mV observed (Fig. 5b). The near overlapping CVs of the 25th and 200th cycles in Fig. 5a show that the current densities are relatively similar, suggesting similar electrochemical activity. In addition, we assessed the stability of FeCo nanocarbide (15% Fe) at a scan rate of 50 mV s^−1^ for 2000 CV cycles and found that a rapid increase of ∼62 mV occurred in the first 500 cycles and remained stable for the rest of the measurement (Fig. S14†). Electrochemical interrogation of FeCo nanocarbide (15% Fe) revealed this sample did not achieve the same magnitude of loss of activity as demonstrated for the lower scan rate, which is corroborated by previous works that observe larger changes in catalyst degradation at low scan rates.^66^

While we investigated the source of the increase in overpotential, other factors were considered which may decrease apparent electrochemical activity, such as the formation of bubbles that block active sites of the electrode surface, physical detachment of the nanomaterial, and hydrophobic/hydrophilic properties of the nanomaterial and the underlying electrode.^67,68^ To avoid some of these deleterious effects, our measurements were monitored by visual inspection every five cycles and large bubbles were removed from the electrode surface when they appeared. Mullins and coworkers showed that their Co_2_C transformed into an amorphous CoO, with an enhancement in OER activity after the first two LSV sweeps.^21^ To determine whether the rapid increase in overpotential, observed in the first 30 cycles for the 15% Fe, was due to oxide reconstruction or other material transformation changes, the materials described in Fig. 5 were analyzed using pXRD before and after OER electrochemical conditioning at 0, 5, 25, 50, 100, and 160 CV cycles (see ESI Fig. S5†). At zero cycles, an oxide layer was not detectable by pXRD, however there is evidence for a partial amorphous oxide layer present using XPS (Fig. S12†). After five cycles, an increase of up to 51% of (Fe_0.15_Co_0.85_)_2_O_4_ was observed (identified by the Fe_2_CoO_4_ reference card) and the material analyzed after 5 or more cycles contributed 51–57% (Fe_0.15_Co_0.85_)_2_O_4_ that remained relatively stable over 5 to 160 cycles (Fig. 5c). Notably, the rapid formation of spinel oxide correlates with the decline of the OER activity (Fig. 5c). The monometallic Co carbide was shown to not have a notable increase in crystalline oxide before and after 30 CV cycles and maintained robust electrochemical stability.^22^ Therefore, we can infer that the initial electrochemical instability in the first 30 CV cycles we observe in the FeCo nanocarbide (15% Fe) resulted from rapid surface oxide formation. To enhance our understanding of the rapid surface reconstruction of 100% carbide to 57% spinel oxide (43% carbide) coverage in the nanomaterial after 160 CV cycles, we further analyzed how much total oxide contributed to the surface layer of the nanoparticle. To determine the surface layer thickness of the (Fe_0.15_Co_0.85_)_2_O_4_ oxide material layer present in the as-synthesized carbide samples, all oxygen atoms were assumed to be present in the surface layer of the particle. We determined that 85% of the total surface layer was attributed to oxide after 160 cycles (more details for calculation in ESI Section 11†), suggesting that a portion of the surface layer is still attributed to carbide and there is not a complete transformation to oxide.

PBA-derived FeCo oxides were synthesized (pXRD shown in ESI Fig. S6†) to better understand the performance of FeCo oxide compared to FeCo carbide electrocatalysts. Both the *in situ* electrochemically oxidized FeCo carbides (post 30 OER cycles) and the PBA-derived FeCo oxides with 15% Fe, resulted in lower electrocatalytic OER activity than the 1^st^ cycle of FeCo carbide. PBA-derived oxide (15% Fe) yielded an average overpotential of 0.70 V at 10 mA cm^−2^ (per geometric area) (Fig. S15†), approximately a 300 mV and 170 mV increase in overpotential in comparison to the *in situ* electrochemically oxidized 15% Fe nanocarbides at the 1^st^ CV cycle (0.41 V) and even the 200^th^ cycle (0.53 V). The 170 mV overpotential difference between the *in situ* electrochemically oxidized nanocarbides and the PBA-derived FeCo oxides could be explained by the difference in the amount of crystalline oxide phase present and possibly the influence of strain on metal surface–oxygen interactions.^69^ Similar cobalt oxide nanocatalysts, such as CoO and Co_3_O_4_, exhibited geometric overpotentials achieved for a current density of 10 mA cm^−2^ of 0.45 V and 0.50 V, respectively,^12^ significantly lower than the 0.70 V achieved for our PBA-derived 15% Fe nanocarbide electrocatalyst. Fe oxide nanocatalysts reported in literature exhibited higher overpotentials than the FeCo carbides, such as 1.23 V at 10 mA cm^−2^ for Fe_2_O_3_,^12^ and 0.45 V at 1 mA cm^−2^ for Fe_3_O_4_.^70^ In contrast to other monometallic Co carbides in literature,^21^ our FeCo carbide electrocatalysts differ in terms of electrocatalytic activity and oxide layer growth. We hypothesize that OER activity can be dependent on material descriptors that result from the harsh oxidative environment, which include the active oxide phase,^71^ phase crystallinity and disorder, and the amount of Fe_2_CoO_4_ oxide present in the surface layer. Previous studies of oxide surface reconstruction have ascribed both rapid OER activity increase^21,72^ and decrease^73^ to the evolution of oxide on the surface during electrocatalytic OER. Oxygen vacancies are another potential phenomenon to occur in reconstructed surface metal oxide catalysts, influencing the local environment and reaction rates of active sites.^74,75^ To relate this to our work, requires a more in-depth analysis of oxygen vacancies on the surface of highly conductive carbon materials with surface oxide reconstruction. Further investigation of stability and oxide surface reconstruction in the carbide family and other non-oxide materials will be fundamental to improving knowledge of designing efficient earth-abundant, non-oxide electrocatalysts for the OER.

## Conclusions

In this work, various ratios of Fe : Co in Fe_*x*_Co_1−*x*_C_*y*_ were controlled through a top-down templated synthetic route, and used to better understand the material composition and structure properties that tune the electrocatalytic activity of bimetallic carbides for the OER. FeCo nanocarbides containing 15–20% Fe resulted in an optimal overpotential of 0.42 V (at 10 mA cm^−2^ per ECSA), with a 100 mV enhancement from the monometallic Co_2_C. Electrochemical stability and material properties of one of the best performing nanocarbides, Fe_0.15_Co_0.85_C_*y*_, were monitored for 200 OER cycles using CV, and a series of samples were analyzed *ex situ* by pXRD. The overpotential achieved increased by ∼110 mV within the first 30 cycles, which was attributed to the growth of an oxide. From this work, it is shown that the Fe_0.15_Co_0.85_C_*y*_ catalyst's oxygen coordinated surface likely undergoes a reconstruction to (Fe_0.15_Co_0.85_)_2_O_4_, that represents 85% of the total surface layer after the first 5 OER CV cycles, and is subsequently maintained. These results support the notion that OER activity is dependent on metal composition and the amount of surface oxide present. Tuning the elemental composition, *i.e.* proportion of Fe and Co present in Fe_*x*_Co_1−*x*_C_*y*_, led to enhanced activity for the OER. This enhanced performance could have resulted from surface level oxide reconstruction, relative surface oxide stabilities, and the amount of oxide layer (post-OER) changing across the composition range. This study provides new insight on the performance of FeCo-based carbide materials for the OER as well as a new strategy for designing multi-metallic carbides as efficient OER electrocatalysts.

## Author contributions

All authors have given approval to the final version of the manuscript.

## Conflicts of interest

There are no conflicts to declare.

## Supplementary Material

RA-013-D3RA07003D-s001
