# Supplementary material for: Electrocatalytic activity and surface oxide reconstruction of bimetallic iron–cobalt nanocarbide electrocatalysts for the oxygen evolution reaction
Source: RSC Adv. 2023 Nov 14;13(47):33413–23. doi: 10.1039/d3ra07003d (PMC10644102; doi:10.1039/d3ra07003d)
Supplement: RA-013-D3RA07003D-s001 [file RA-013-D3RA07003D-s001.pdf]

Electronic Supplementary Material (ESI) for RSC Advances.
This journal is © The Royal Society of Chemistry 2023

Supporting Information for article, “Electrocatalytic activity and surface oxide reconstruction of bimetallic iron-cobalt nanocarbide electrocatalysts for the oxygen evolution reaction”

*Amanda J. Ritz‡, Isabella A. Bertini‡, Edward T. Nguyen, Geoffrey F. Strouse, Robert A. Lazenby**

Department of Chemistry & Biochemistry, Florida State University, Tallahassee, Florida

Section 1. SEM images of FeCo Prussian blue analogue (PBA) precursors. 2

Section 2. Powder X-ray diffraction of PBA precursors. 3

Section 3. X-ray fluorescence elemental composition for FeCo PBA precursors and FeCo carbides. 4

Section 4. Powder X-ray diffraction and fits for $Fe_xCo_{1-x}C_y$ composition range. 5

Section 5. Powder XRD 15% Fe FeCo post OER cycling. 7

Section 6. Powder XRD of Fe, Co and 15% Fe FeCo oxides. 8

Section 7. SEM micrograph of drop casted nanomaterial modified glassy carbon surface. 9

Section 8. Representative double-layer capacitance measurements for determining electrochemically active surface area for an FeCo nanocarbide. 10

Section 9. OER polarization curves with iR compensation. 11

Section 10. Additional voltammetry data on FeCo nanocarbides. 11

Section 11. Overpotential comparisons of bimetallic OER catalysts from literature. 13

Section 12. XPS chemical shifts, and calculation of oxide percentage for select $Fe_xCo_{1-x}C_y$ samples. 14

Section 13. Determination of surface layer thickness. 16

Section 14. Electrochemical stability of commercial RuO2 nanoparticles. 17

Section 15. Additional stability measurements of FeCo nanocarbides. 17

Section 16. Linear sweep voltammograms (LSVs) of PBA-derived 15% Fe containing FeCo oxide. 18

References 19

Section 1. SEM images of FeCo Prussian blue analogue (PBA) precursors.

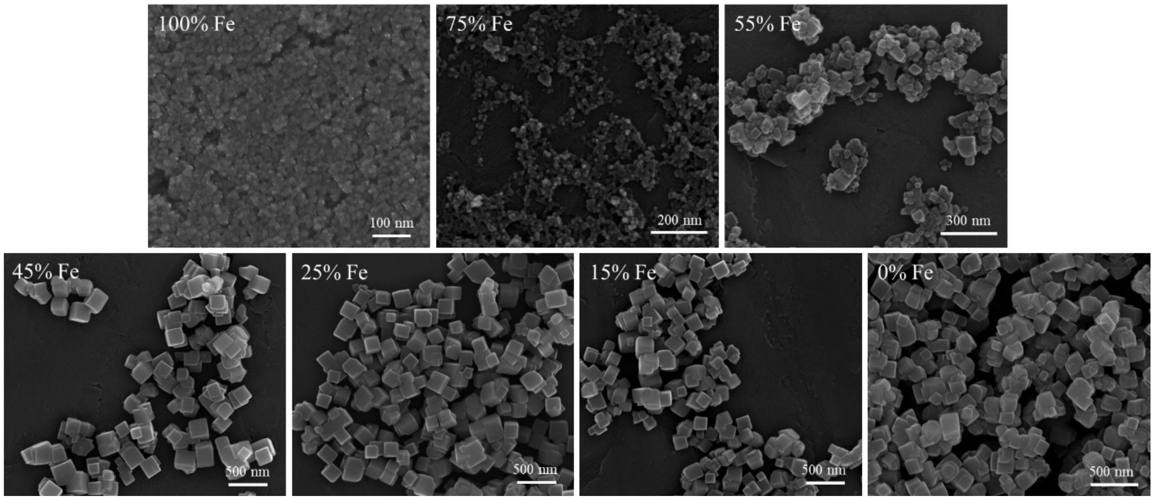

Figure S1. Scanning electron microscopy (SEM) images of FeCo Prussian blue analogue (PBA) precursors with increasing % Fe. Sizes range from < 20 nm – 150 nm.

Section 2. Powder X-ray diffraction of PBA precursors.

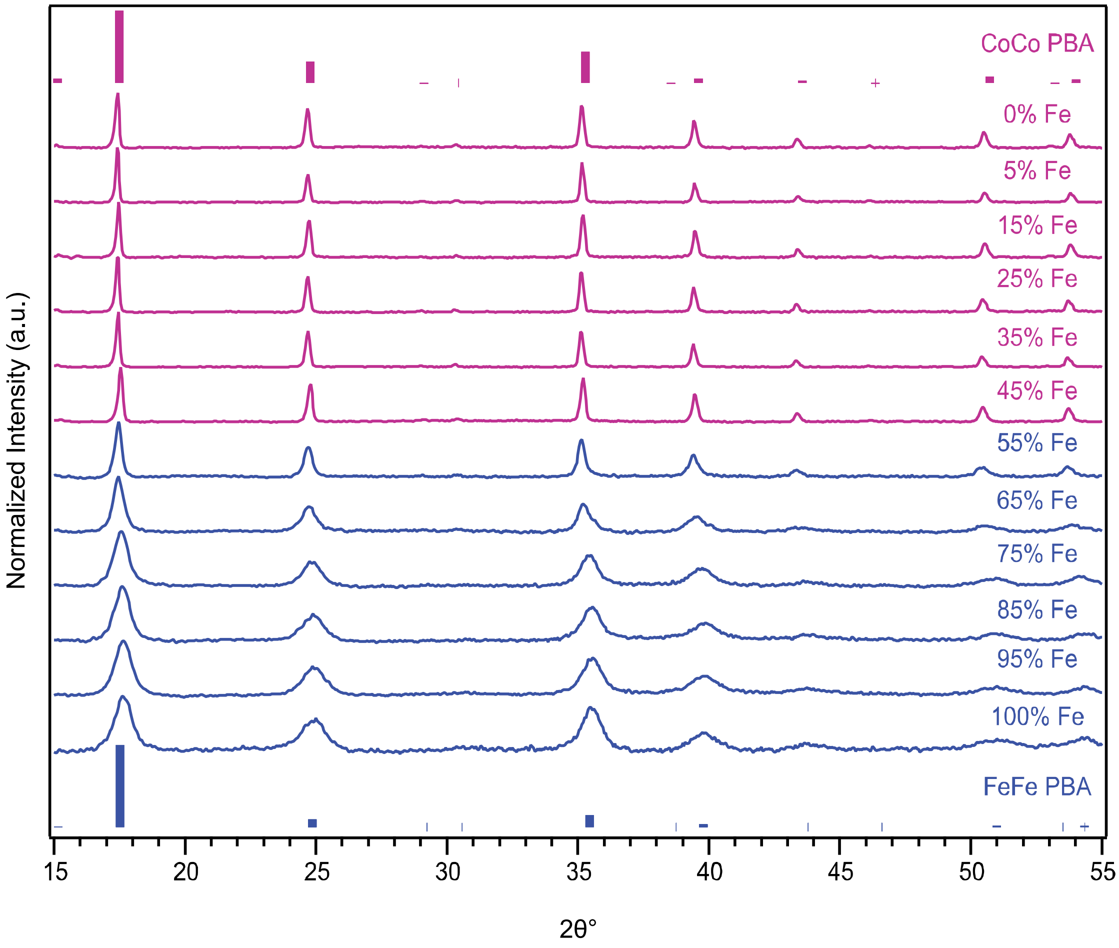

Figure S2. Powder X-ray diffraction patterns of FeCo PBA precursors. A transition from the $KCoCo(CN)_6 \cdot x\ H_2O$ PBA type to the $KFeFe(CN)_6 \cdot x\ H_2O$ is observed as the amount of Fe increases, corresponding to a Vegard shift towards lower 2θ and the space group of Fm-3m remains for both PBA forms. The pink reference card (ICSD 45154) represents the $KCoCo(CN)_6 \cdot x\ H_2O$ type and the blue reference card (ICSD 23102) represents the $KFeFe(CN)_6 \cdot x\ H_2O$ type.

Section 3. X-ray fluorescence elemental composition for FeCo PBA precursors and FeCo carbides.

Table S1. X-ray fluorescence (XRF) elemental composition for FeCo PBA precursors and FeCo carbides. Results show ratio of metals are maintained from precursor to resultant carbide.

Sample (PBA Precursors)	% Fe	% Co
100% Fe	99	1
95% Fe	93	7
85% Fe	95	5
75% Fe	84	16
65% Fe	72	28
55% Fe	60	40
45% Fe	33	67
35% Fe	26	74
25% Fe	18	82
20% Fe	14	86
15% Fe	12	88
5% Fe	7	93
0% Fe	0	100

Sample (FeCo Carbide)	% Fe	% Co
100% Fe	100	0
95% Fe	93	7
85% Fe	95	5
75% Fe	80	20
65% Fe	71	29
55% Fe	60	40
45% Fe	35	65
35% Fe	27	73
25% Fe	20	80
20% Fe	14	86
15% Fe	12	88
5% Fe	4	96
0% Fe	0	100

Section 4. Powder X-ray diffraction and fits for $Fe_xCo_{1-x}C_y$ composition range.

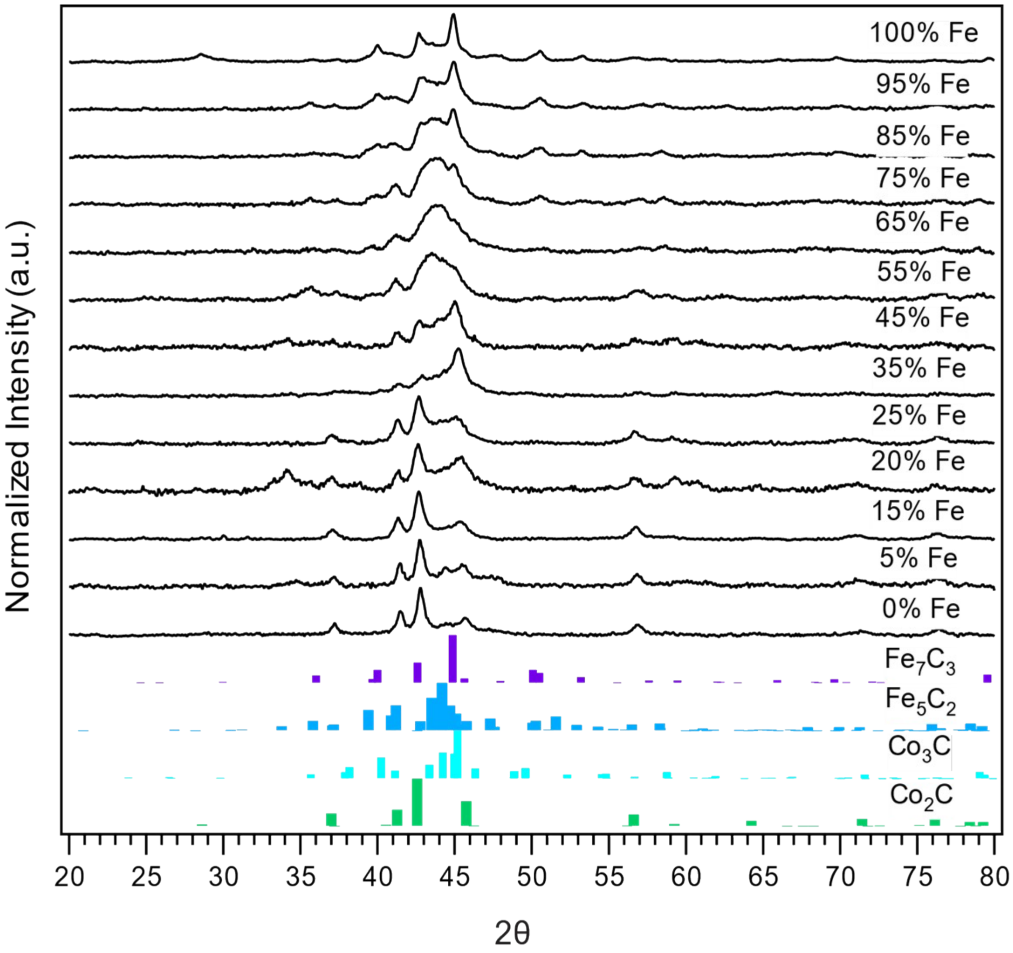

Figure S3. Powder X-ray diffraction (pXRD) patterns of all $Fe_xCo_{1-x}C_y$ samples produced for electrocatalytic study, of varying Fe content from 0 – 100%. Pure reference phases are shown for comparison, for M_2C (Green reference, COD: 1528415), M_3C (light blue reference, ICSD: 43521), M_5C_2 (dark blue reference, ICSD: 423885) and M_7C_3 (purple reference, ICSD: 76830).

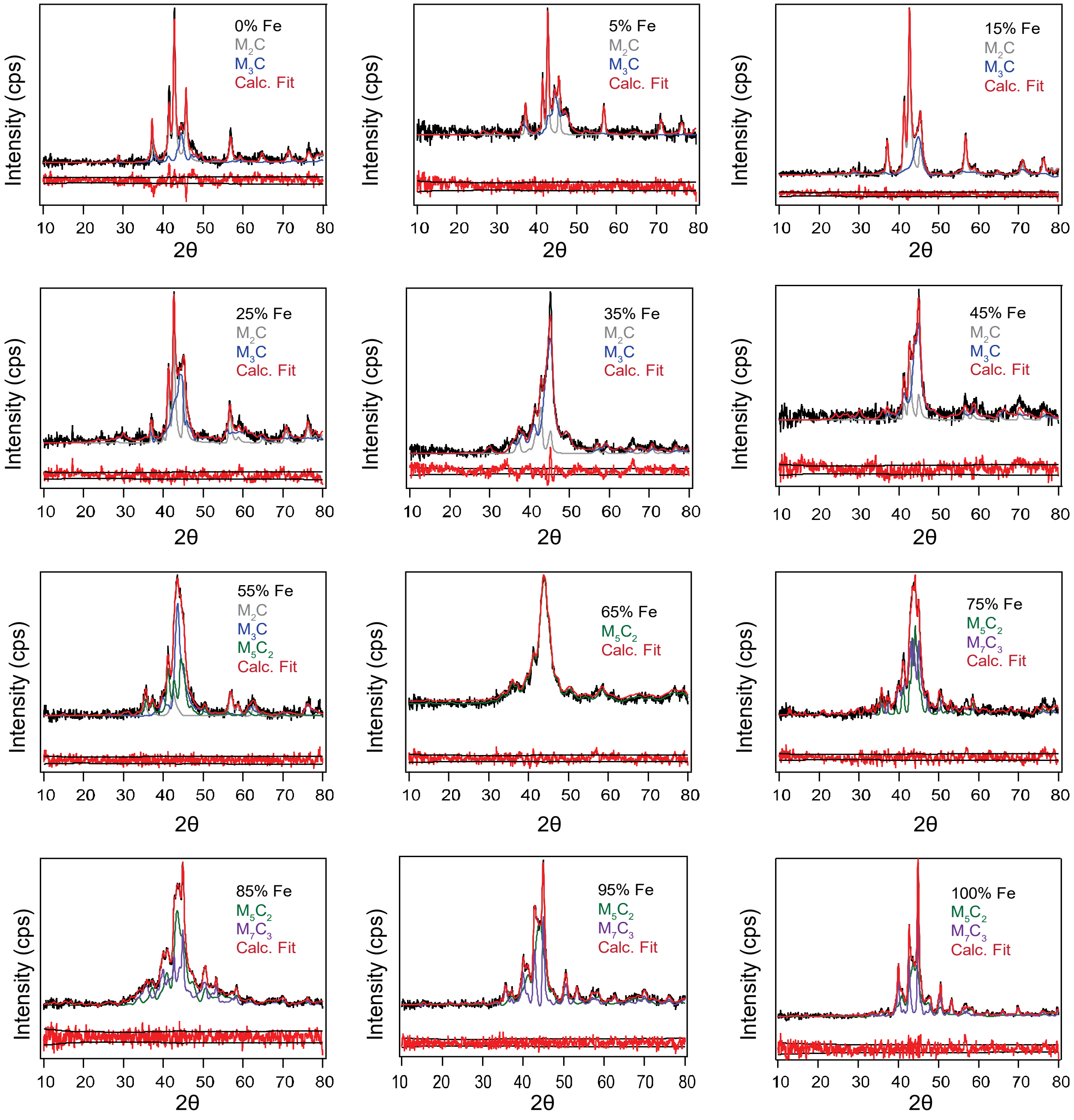

Figure S4. Contribution of varying phases fitted (upper red) to each $Fe_xCo_{1-x}C_y$ powder XRD pattern. Residuals are plotted below each fit (lower red).

Section 5. Powder XRD 15% Fe FeCo post OER cycling.

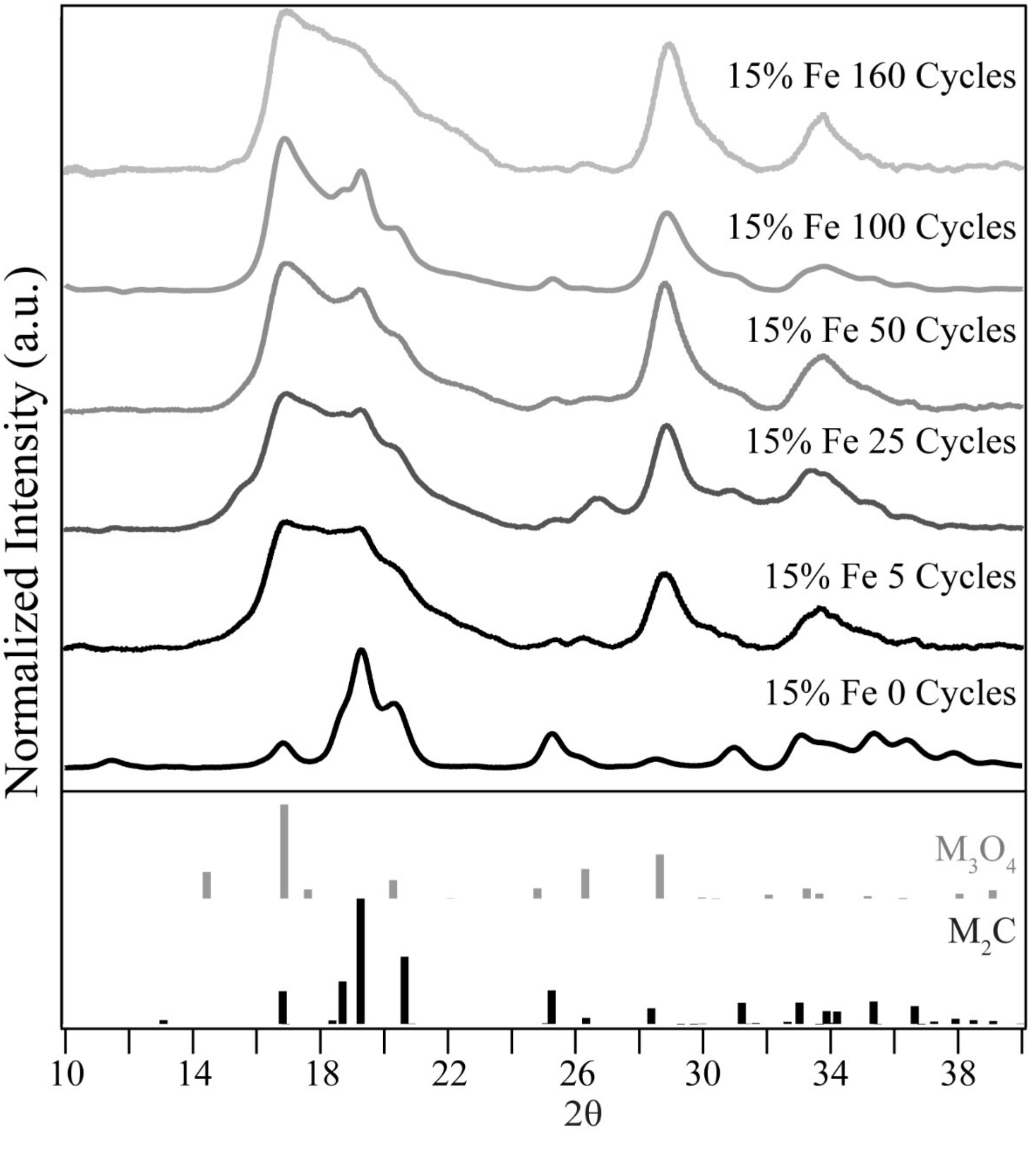

Figure S5. Powder X-ray diffraction of the 15% Fe sample, post OER cycling study. pXRD reference cards shown below, in grey M_3O_4 (Fe_2CoO_4 structure) and in black M_2C (Co_2C structure). The intensities of the M_2C peaks, I_{M2C}, are normalized to 1 in each spectrum The percentage of M_3O_4 in the sample was estimated using the relative intensities of the most prominent pXRD peak for M_3O_4 (16.88 2θ) and M_2C (19.26 2θ) using:

$$\% M_3O_4 = (I_{M3O4}/(I_{M3O4} + I_{M2C})) \times 100$$

Section 6. Powder XRD of Fe, Co and 15% Fe FeCo oxides.

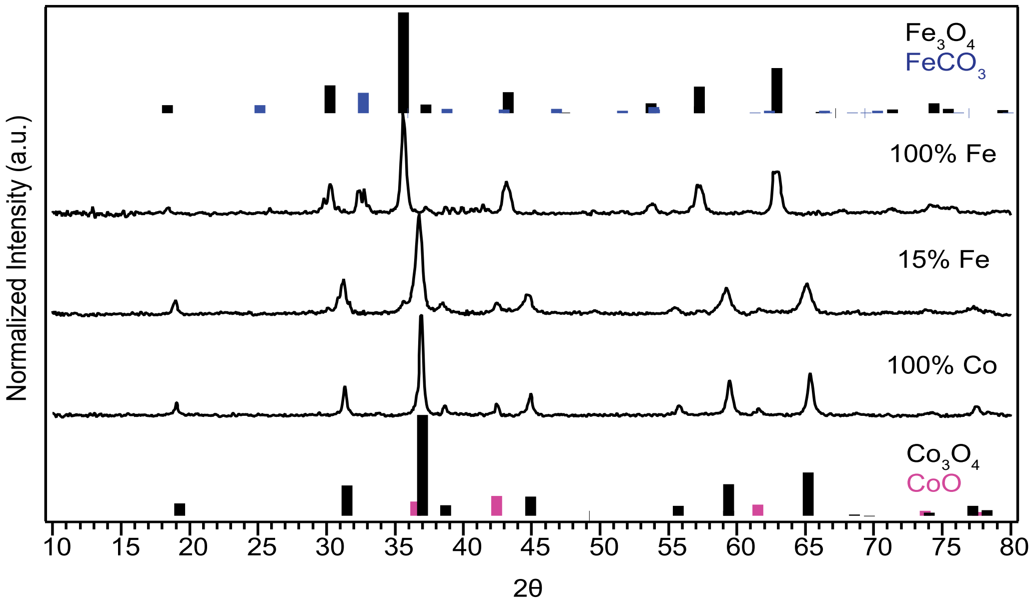

Figure S6. Powder XRD patterns of FeCo oxides, for 0% Fe (i.e. 100% Co), 15% Fe and 100% Fe. Pure phase references are shown for comparison for Co_3O_4 (bottom black, reference ICSD 24210), CoO (bottom pink, reference ICSD 174027), Fe_3O_4 (top blue, reference ICSD 75627) and $FeCO_3$ (top black, reference COD 9014728).

Section 7. SEM micrograph of drop casted nanomaterial modified glassy carbon surface.

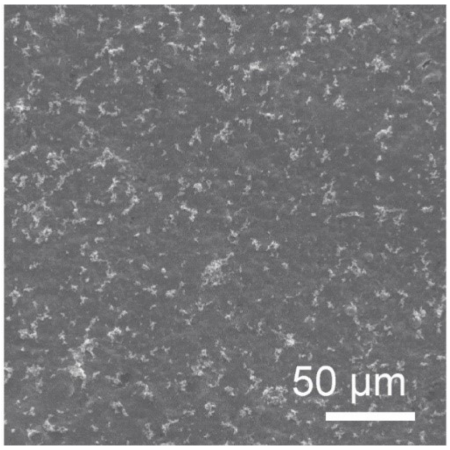

Figure S7. SEM micrograph of a homogenously drop casted FeCo nanocarbide, containing 55% Fe, on a glassy carbon wafer electrode.

Section 8. Representative double-layer capacitance measurements for determining electrochemically active surface area for an FeCo nanocarbide.

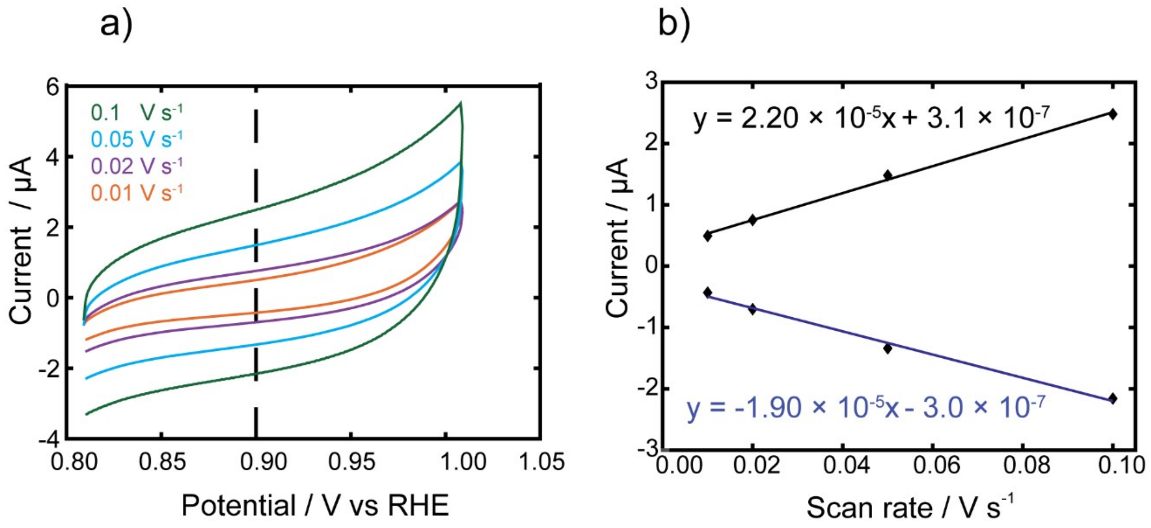

Figure S8. Representative double-layer capacitance measurements for determining electrochemically active surface area for a 45% Fe containing FeCo nanocarbide. a) Cyclic voltammograms (CVs) were measured in a non-Faradaic current region, using scan rates of 0.01, 0.02, 0.05, and 0.10 V s^{-1}. The cathodic and anodic charging currents were both measured at a fixed potential of 0.9 V vs. RHE (shown by dashed line), and are shown as a plot of current vs. scan rate in b) with the resulting slopes representing the double layer capacitance used to calculate electrochemical surface area.

Section 9. OER polarization curves with iR compensation.

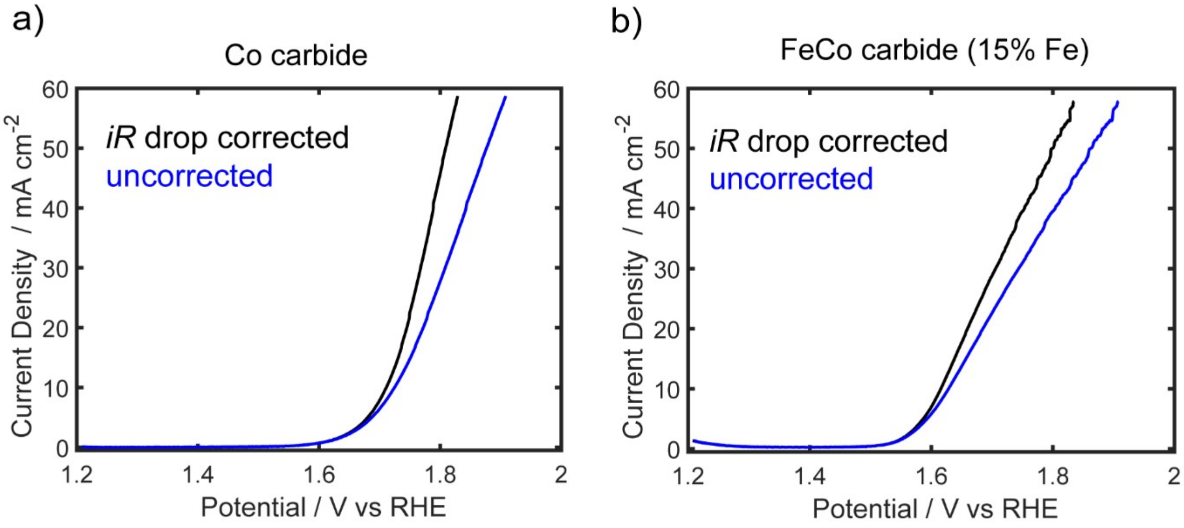

Figure S9. OER polarization curves of iR drop corrected (black) and uncorrected (blue) curves for a) Co carbide and b) FeCo carbide (15% Fe).

Section 10. Additional voltammetry data on FeCo nanocarbides.

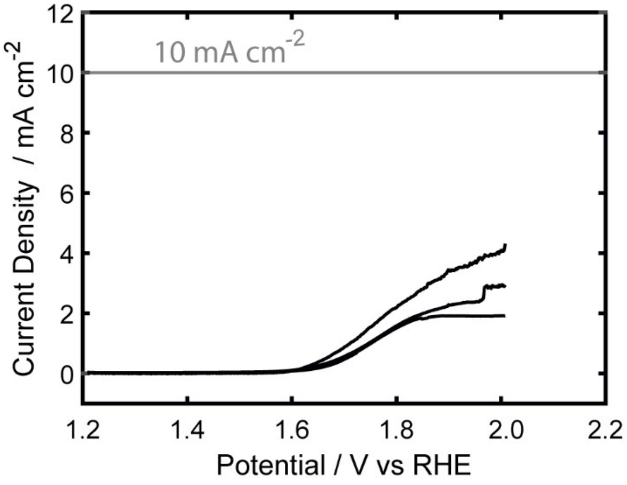

Figure S10. Three linear sweep voltammograms of monometallic Fe carbide samples drop cast on glassy carbon electrodes in 1 M KOH. Samples did not achieve a current density of 10 mA cm^{-2}.

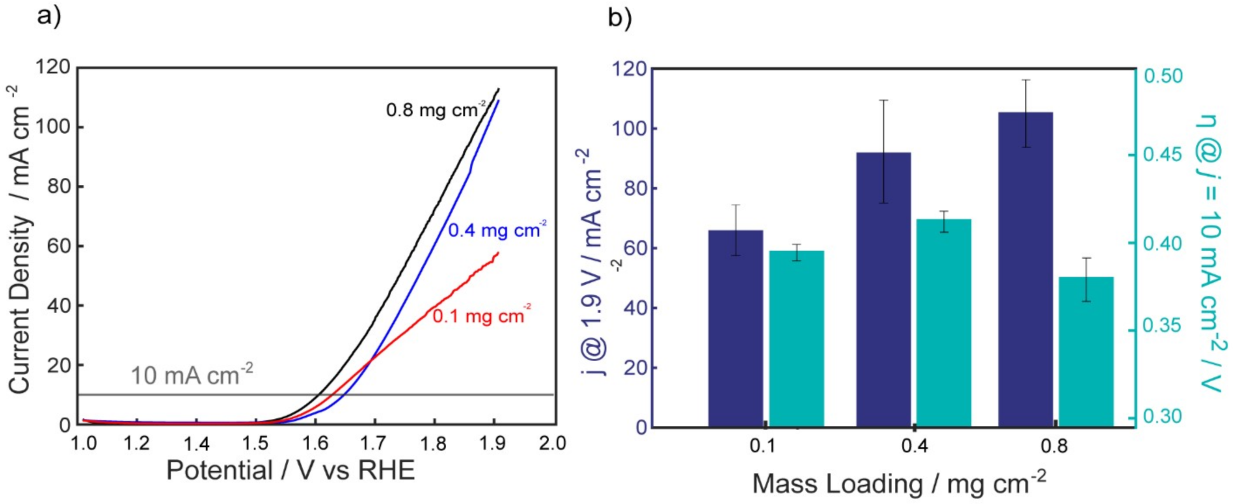

Figure S11. a) Linear sweep voltammograms in 1 M KOH were collected of FeCo nanocarbide (15% Fe) drop cast on a glassy carbon electrode at varying mass loadings of 0.1 mg cm^{-2} (red), of 0.4 mg cm^{-2} (blue), and 0.8 mg cm^{-2} (black). Current densities extracted at 1.9 V and overpotentials extracted at a current density of 10 mA cm^{-2} were plotted as a bar graph in b) at varying mass loadings.

Section 11. Overpotential comparisons of bimetallic OER catalysts from literature.

Table S-2. Overpotential comparisons of bimetallic FeNi, FeCo, and other bimetallic catalyst systems in alkaline conditions for OER electrocatalytic activity.

**Electrocatalyst**	**Optimum ratio (X:X)**	**Support / substrate**	**Mass loading / mg cm^{-2}**	**η_{geo}@ 10 mA cm^{-2} / V**	**Reference**
**Noble metal**					
**RuO_2**	**--**	**GCE**	**0.10**	**0.36**	**This work**
RuO_2	--	GCE	Sputtered	0.38	1
IrO_2	--	GCE	Sputtered	0.38	1
**FeCo**					
**$Fe_xCo_{1-x}C_y$**	**20:80**	**GCE**	**0.10**	**0.40**	**This work**
$(Co_xFe_{1-x})_2P$	1:1	GCE	--	0.37	2
FeCoOOH/ NS	1:1	NF	--	0.21	3
FeCo/C NS	1:1	NF	--	0.22	4
α-$CoFe(OH)_2$	4:1	Ti	0.28	0.30	5
Spinel Co_4FeO	4:1	Ti	0.28	0.40	5
FeCo-LDH NS	1:1	GCE	0.70	0.28	6
**FeNi**					
$Fe_xNi_{1-x}O$	10:90	Au	-- / A = 0.196 cm^2	0.28	7
NiFeOOH	65:35	Graphene	-- / A = 0.025 cm^2	0.32	8
$NiFe(OH)_2$	4:1	Au	0.14	0.26	9
$NiFeO_x$	9:1	Au/Ti	--	0.34	10
$NiFe(OH)_2$	2:1	Graphene (oxide)	0.25	0.23	11
$NiFeO_x$/C	69:31	GCE	--	0.28	12
**Other bimetallic systems**					
$Co_6Mo_6C_2$	--	NCRGO	0.14	0.26	13
Mo_6Ni_6C	--	NF	9.8	0.19	14
Co_6W_6C	--	CC	7 – 10	0.29	15
Fe: Ni_3C	2:98	GCE	0.15	0.28	16
Co: Ni_3C	1:1	Ni@C	0.20	0.22	17
Fe_3Mo_3C	--	GCE	0.25	~0.67	18
Fe/Co:NC	1:1	GCE	0.30	1.6	19

GCE = Glassy carbon electrode, Au = gold, IF = Iron foam, NF = Nickel foam, NS = nanosheet, LDH = layered double hydroxides, A = electrochemically active area, RF = roughness factor, NCRGO = N-doped reduced graphene oxide.

Section 12. XPS chemical shifts, and calculation of oxide percentage for select $Fe_xCo_{1-x}C_y$ samples.

Table S3. Table of all XPS chemical shifts for selected $Fe_xCo_{1-x}C_y$ samples. All chemical shifts were calibrated to C1s = 284.8 eV. No clear trend is correlative to experimental electrocatalysis results, and minimal changes are seen in the overall electronic environment.

Sample	Co Chemical Shifts (eV)	Fe Chemical Shifts (eV)	C Chemical Shifts (eV)	O Chemical Shifts (eV)
0% Fe	Co^0: 778.5<br>Co^{2+}: 782.3<br>Co^{3+}: 780.0	N/A	C-M: 284.8<br>C-C: 285.8<br>C-O: 287.6	O-Fe: 530.3<br>O-Co: 531.6<br>O-C: 532.7
15% Fe	Co^0: 778.3<br>Co^{2+}: 782.7<br>Co^{3+}: 780.3	Fe^0: 707.0<br>Fe^{2+}: 709.3<br>Fe^{3+}: 711.3	M-C: 284.8<br>C-C: 286.6<br>C-O: 288.9	O-Fe: 530.1<br>O-Co: 531.8<br>O-C: 532.7
25% Fe	Co^0: 778.2<br>Co^{2+}: 781.4<br>Co^{3+}: 779.1	Fe^0: 707.5<br>Fe^{2+}: 709.9<br>Fe^{3+}: 711.8	M-C: 284.8<br>C-C: 286.1<br>C-O: 288.2	O-Fe: 530.4<br>O-Co: 532.1<br>O-C: 535.2
45% Fe	Co^0: 777.9<br>Co^{2+}: 781.8<br>Co^{3+}: 779.5	Fe^0: 706.7<br>Fe^{2+}: 708.8<br>Fe^{3+}: 710.7	M-C: 284.8<br>C-C: 284.9<br>C-O: 287.2	O-Fe: 529.9<br>O-Co: 531.6<br>O-C: 533.2
55% Fe	Co^0: 778.3<br>Co^{2+}: 783.3<br>Co^{3+}: 780.1	Fe^0: 707.1<br>Fe^{2+}: 709.3<br>Fe^{3+}: 711.2	M-C: 284.8<br>C-C: 286.5<br>C-O: 288.5	O-Fe: 530.3<br>O-Co: 532.0<br>O-C: 533.4
75% Fe	Co^0: 778.6<br>Co^{2+}: 786.0<br>Co^{3+}: 782.3	Fe^0: 707.1<br>Fe^{2+}: 709.3<br>Fe^{3+}: 711.2	M-C: 284.8<br>C-C: 286.6<br>C-O: 288.7	O-Fe: 530.3<br>O-Co: 532.0<br>O-C: 533.4
100% Fe	---	Fe^0: 707.3<br>Fe^{2+}: 709.5<br>Fe^{3+}: 711.2	M-C: 284.8<br>C-C: 285.3<br>C-O: 287.3	O-Fe: 530.4<br>O-Co: 532.1<br>O-C: 533.6

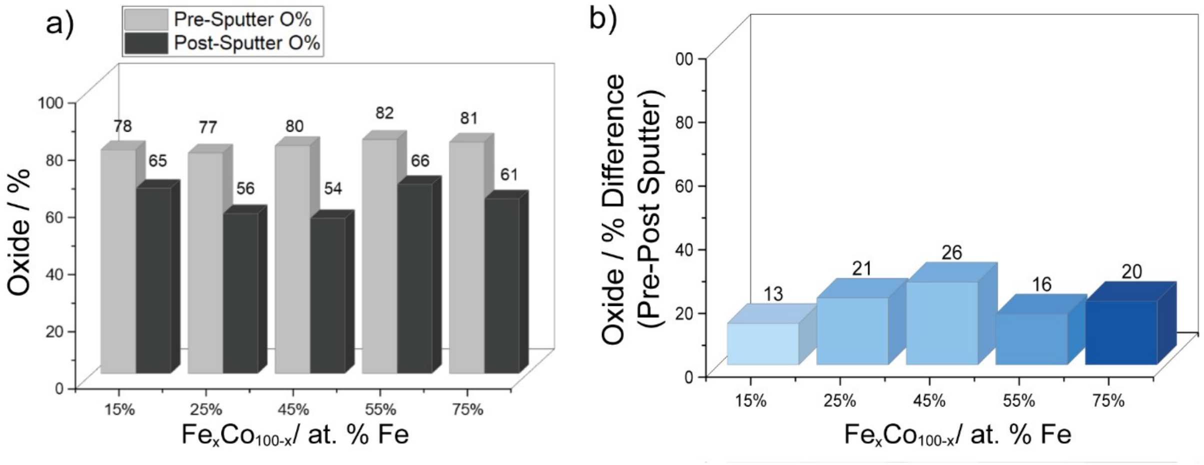

Figure S12. a) XPS quantification of oxide present in as synthesized $Fe_xCo_{1-x}C_y$ samples before (light gray) and after (dark gray) Ar ion sputtering at 5 keV/1 μA for 15 minutes. Note that carbon atomic percentages were not included as samples were run on a carbon puck, therefore the relative amount of only metal and oxygen can be accurately observed. b) Percent difference of oxide in various $Fe_xCo_{1-x}C_y$ samples. Interestingly, 15% shows the smallest oxide percent difference suggesting that minor oxide surface layers may enhance electrocatalysis.

Section 13. Determination of surface layer thickness.

To determine surface layer thickness, first, the volume, *V*, of the carbide nanoparticle was calculated to be 382 nm^3 for a 9 nm (diameter) particle using

$$V = \frac{4}{3}\pi r^3 \quad (1)$$

where *r* is the radius of the nanoparticles. Then, the surface area, *SA*, of the particle was calculated to be 254 nm^2 using

$$SA = 4\,\pi r^2$$
(2)

Therefore, the surface area to volume ratio of the nanoparticle is 67% using

$$SA\ to\ V\ ratio\ in\ percent\ form = \frac{SA}{V} \times 100 \quad (3)$$

Assuming 67% of the total volume or 254 nm^3 constitutes the surface layer, an estimation of the particle's core volume was calculated as 128 nm^3 by taking 33% of the total particle volume. The radius of the core, r_C, is then 3.11 nm, using

$$r_C = \sqrt[3]{\frac{128 \times 3}{4\pi}} \quad (4)$$

The difference of the total radius of the particle, r_T, and the radius of the core, r_C, is equal to the thickness of the surface layer, *t*, which equals 13.9 Å using

$$t = r_T - r_C$$
(5)

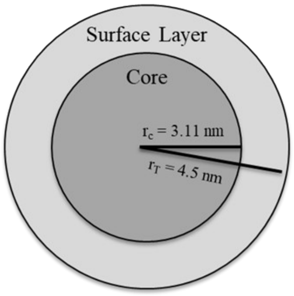

The material after 160 OER CV cycles contained up to 85% total oxide present in the surface layer. This was calculated as shown below:

$$total\ oxide\ in\ surface\ layer = \frac{57\ (percent\ of\ total\ oxide\ in\ particle\ )}{67\ (percent\ of\ surface\ layer\ volume)} \times 100 \quad (6)$$

Section 14. Electrochemical stability of commercial RuO2 nanoparticles.

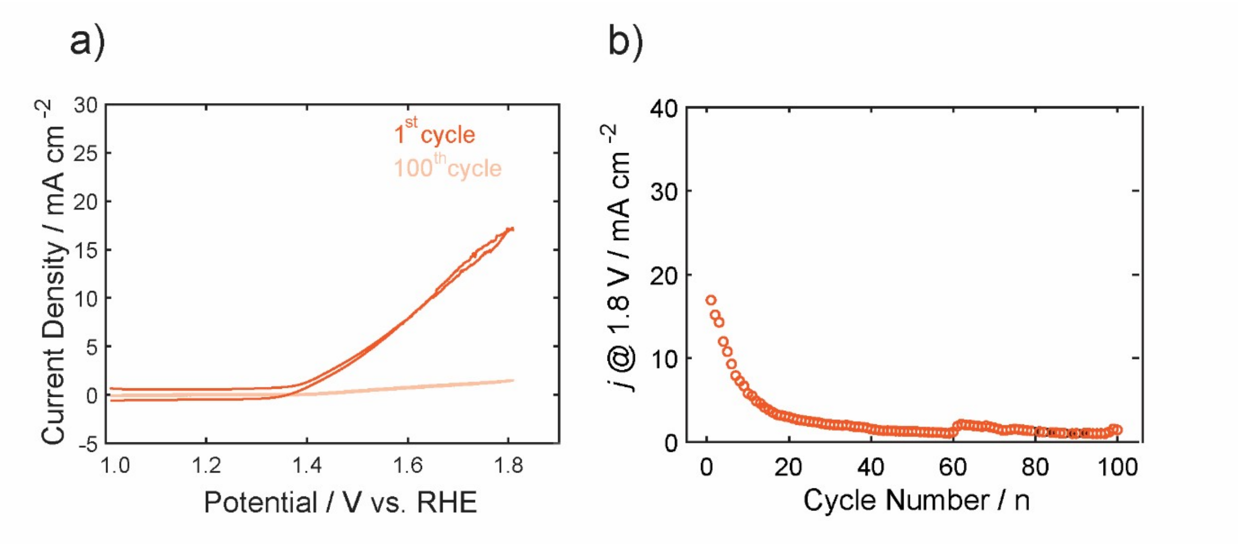

Figure S13. a) CVs of the 1st and 100th cycle of RuO_2 at a scan rate of 5 mV s^{-1}, and b) the decay of current density at a maximum potential of 1.8 V vs. RHE over the 100 CV cycles.

Section 15. Additional stability measurements of FeCo nanocarbides.

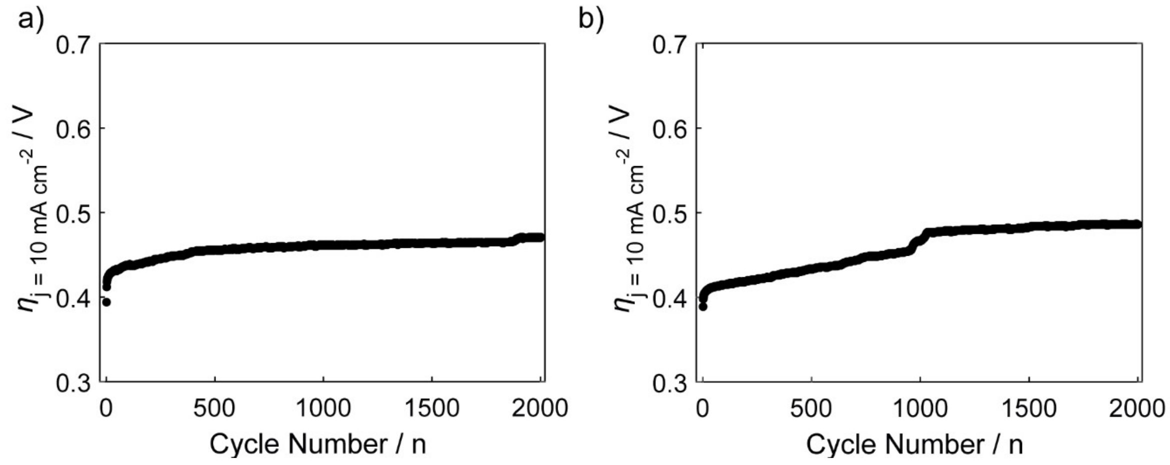

Figure S14. Stability measurement of FeCo nanocarbide (15% Fe) with overpotentials extracted from a current density of 10 mA cm^{-2}, collected at a scan rate of 50 mV s^{-1} in 1 M KOH with a) representing the first measurement and b) the second measurement collected.

Section 16. Linear sweep voltammograms (LSVs) of PBA-derived 15% Fe containing FeCo oxide.

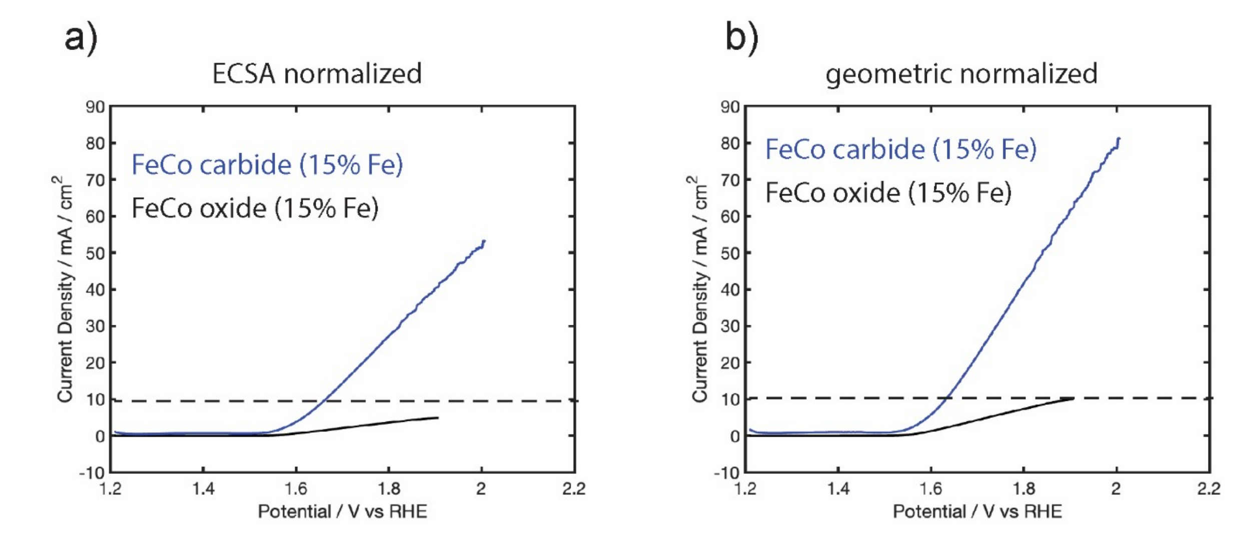

Figure S15. Linear sweep voltammograms (LSVs) of PBA-derived FeCo oxide (15% Fe), compared to FeCo nanocarbide (15% Fe) with a) ECSA normalized and b) geometric normalized current densities.

References

(1) Jung, S.; McCrory, C. C. L.; Ferrer, I. M.; Peters, J. C.; Jaramillo, T. F. Benchmarking Nanoparticulate Metal Oxide Electrocatalysts for the Alkaline Water Oxidation Reaction. *J. Mater. Chem. A* **2016**, *4* (8), 3068–3076. https://doi.org/10.1039/c5ta07586f.

(2) Mendoza-Garcia, A.; Su, D.; Sun, S. Sea Urchin-like Cobalt-Iron Phosphide as an Active Catalyst for Oxygen Evolution Reaction. *Nanoscale* **2016**, *8* (6), 3244–3247. https://doi.org/10.1039/c5nr08763e.

(3) Nguyen, T. T. H.; Lee, J.; Bae, J.; Lim, B. Binary FeCo Oxyhydroxide Nanosheets as Highly Efficient Bifunctional Electrocatalysts for Overall Water Splitting. *Chem. - A Eur. J.* **2018**, *24* (18), 4724–4728. https://doi.org/10.1002/chem.201800022.

(4) Xiang, R.; Duan, Y.; Tong, C.; Peng, L.; Wang, J.; Shah, S. S. A.; Najam, T.; Huang, X.; Wei, Z. Self-Standing FeCo Prussian Blue Analogue Derived FeCo/C and FeCoP/C Nanosheet Arrays for Cost-Effective Electrocatalytic Water Splitting. *Electrochim. Acta* **2019**, *302*, 45–55. https://doi.org/10.1016/j.electacta.2019.01.170.

(5) Jin, H.; Mao, S.; Zhan, G.; Xu, F.; Bao, X.; Wang, Y. Fe Incorporated α-Co(OH)2 Nanosheets with Remarkably Improved Activity towards the Oxygen Evolution Reaction. *J. Mater. Chem. A* **2017**, *5* (3), 1078–1084. https://doi.org/10.1039/c6ta09959a.

(6) Qiao, X.; Kang, H.; Wu, J.; Li, Y.; Wang, Q.; Jia, X.; Qiao, Y.; Lu, S.; Wu, X.; Qin, W. A Partial Sulfidation Approach That Significantly Enhance the Activity of FeCo Layered Double Hydroxide for Oxygen Evolution Reaction. *Int. J. Hydrogen Energy* **2019**, *44* (60), 31987–31994. https://doi.org/10.1016/j.ijhydene.2019.10.082.

(7) Mu, A.; Scheu, C.; Pokharel, A.; Bo, S.; Bein, T.; Fattakhova-rohlfing, D. Iron-Doped Nickel Oxide Nanocrystals Alkaline Water Splitting. *ACS Nano* **2015**, *9* (5), 5180–5188.

(8) Görlin, M.; Halldin Stenlid, J.; Koroidov, S.; Wang, H. Y.; Börner, M.; Shipilin, M.; Kalinko, A.; Murzin, V.; Safonova, O. V.; Nachtegaal, M.; Uheida, A.; Dutta, J.; Bauer, M.; Nilsson, A.; Diaz-Morales, O. Key Activity Descriptors of Nickel-Iron Oxygen Evolution Electrocatalysts in the Presence of Alkali Metal Cations. *Nat. Commun.* **2020**, *11* (1), 1–11. https://doi.org/10.1038/s41467-020-19729-2.

(9) Yu, X.; Zhang, M.; Yuan, W.; Shi, G. A High-Performance Three-Dimensional Ni-Fe Layered Double Hydroxide/Graphene Electrode for Water Oxidation. *J. Mater. Chem. A* **2015**, *3* (13), 6921–6928. https://doi.org/10.1039/c5ta01034a.

(10) Trotochaud, L.; Ranney, J. K.; Williams, K. N.; Boettcher, S. W. Solution-Cast Metal Oxide Thin Film Electrocatalysts for Oxygen Evolution. *J. Am. Chem. Soc.* **2012**, *134* (41), 17253–17261. https://doi.org/10.1021/ja307507a.

(11) Ma, W.; Ma, R.; Wang, C.; Liang, J.; Liu, X.; Zhou, K.; Sasaki, T. A Superlattice of

Alternately Stacked Ni-Fe Hydroxide Nanosheets and Graphene for Efficient Splitting of Water. *ACS Nano* **2015**, *9* (2), 1977–1984. https://doi.org/10.1021/nn5069836.

(12) Qiu, Y.; Xin, L.; Li, W. Electrocatalytic Oxygen Evolution over Supported Small Amorphous Ni-Fe Nanoparticles in Alkaline Electrolyte. *Langmuir* **2014**, *30* (26), 7893–7901. https://doi.org/10.1021/la501246e.

(13) Tang, Y. J.; Liu, C. H.; Huang, W.; Wang, X. L.; Dong, L. Z.; Li, S. L.; Lan, Y. Q. Bimetallic Carbides-Based Nanocomposite as Superior Electrocatalyst for Oxygen Evolution Reaction. *ACS Appl. Mater. Interfaces* **2017**, *9* (20), 16977–16985. https://doi.org/10.1021/acsami.7b01096.

(14) Zu, M. Y.; Wang, C.; Zhang, L.; Zheng, L. R.; Yang, H. G. Reconstructing Bimetallic Carbide Mo6Ni6C for Carbon Interconnected MoNi Alloys to Boost Oxygen Evolution Electrocatalysis. *Mater. Horizons* **2019**, *6* (1), 115–121. https://doi.org/10.1039/C8MH00664D.

(15) Chen, J.; Ren, B.; Cui, H.; Wang, C.; Chen, J.; Ren, B.; Cui, H.; Wang, C. Constructing Pure Phase Tungsten-Based Bimetallic Carbide Nanosheet as an Efficient Bifunctional Electrocatalyst for Overall Water Splitting. *Small* **2020**, *16* (23), 1907556. https://doi.org/10.1002/SMLL.201907556.

(16) Fan, H.; Yu, H.; Zhang, Y.; Zheng, Y.; Luo, Y.; Dai, Z.; Li, B.; Zong, Y.; Yan, Q. Fe-Doped Ni3C Nanodots in N-Doped Carbon Nanosheets for Efficient Hydrogen-

Evolution and Oxygen-Evolution Electrocatalysis. *Angew. Chemie Int. Ed.* **2017**, *56* (41), 12566–12570. https://doi.org/10.1002/ANIE.201706610.

(17) Jia, X.; Wang, M.; Liu, G.; Wang, Y.; Yang, J.; Li, J. Mixed-Metal MOF-Derived Co-Doped Ni3C/Ni NPs Embedded in Carbon Matrix as an Efficient Electrocatalyst for Oxygen Evolution Reaction. *Int. J. Hydrogen Energy* **2019**, *44* (45), 24572–24579. https://doi.org/10.1016/J.IJHYDENE.2019.07.144.

(18) Cui, Z.; Li, Y.; Fu, G.; Li, X.; Goodenough, J. B.; Cui, Z.; Li, Y.; Fu, G.; Li, X.; Goodenough, B. Robust Fe3Mo3C Supported IrMn Clusters as Highly Efficient Bifunctional Air Electrode for Metal–Air Battery. *Adv. Mater.* **2017**, *29* (40), 1702385. https://doi.org/10.1002/ADMA.201702385.

(19) Yang, J.; Wang, X.; Li, B.; Ma, L.; Shi, L.; Xiong, Y.; Xu, H. Novel Iron/Cobalt-Containing Polypyrrole Hydrogel-Derived Trifunctional Electrocatalyst for Self-Powered Overall Water Splitting. *Adv. Funct. Mater.* **2017**, *27* (17), 1606497. https://doi.org/10.1002/adfm.201606497.
